# Synchronized by photoperiod: Shedding light on flowering seasonality in *Acrocomia* species (Arecaceae)

**DOI:** 10.1371/journal.pone.0352981

**Published:** 2026-07-16

**Authors:** Catherine Jeanne Marguerite Meyer, Cláudio Emanuel Magaton, Thomas Hilger, Kalcida Naomi Kuki, Sérgio Yoshimitsu Motoike, Georg Cadisch

**Affiliations:** 1 Hans-Ruthenberg-Institute for Tropical Agricultural Sciences, University of Hohenheim, Stuttgart, Germany; 2 Department of Forest Engineering, Federal University of Viçosa, Viçosa, Brazil; 3 Department of Agronomy, Federal University of Viçosa, Viçosa, Brazil; Institute for Biological Research, University of Belgrade, SERBIA

## Abstract

Interest in the oilseed palm genus *Acrocomia* has grown due to its potential as a sustainable alternative to the African oil palm (*Elaeis guineensis*). Recent research has focused on genetic resource development, and oil yield and quality, and applying this knowledge toward domestication. However, major knowledge gaps remain regarding yield formation, particularly the understanding of flowering phenology. In addition, the environmental drivers regulating its flowering phenology remain unclear. While flowering is often assumed to be triggered by the onset of the rainy season, evidence from different regions suggests that this cue is unreliable. This study aimed to determine the climatic and photoperiodic drivers of *Acrocomia* flowering seasonality and to assess variation among accessions of *A. totai*, *A. intumescens* and *A. aculeata* from diverse South American origins. We monitored six *Acrocomia* accessions cultivated under identical conditions in the Active Germplasm Bank of *Acrocomia* (BAG-Macaúba) in Araponga, Brazil, over three flowering seasons (2019–2021). Using circular statistics, seasonal decomposition, and moving-window correlations, we analyzed relationships between flowering patterns and temperature, precipitation, solar radiation, and photoperiod. The flowering exhibited strong seasonality from September to January, with synchronous patterns across seasons. Photoperiod was identified as the predominant factor associated with flowering timing, while precipitation and temperature showed weaker and less consistent links, contributing only to minor interannual fluctuations. These results suggest that photoperiod may act as a primary seasonal cue for *Acrocomia* lowering, while episodic climatic variations act as secondary modukators. This improved comprehension facilitates the formulation of strategies for pollination management, genotype selection, and yield stability in prospective *Acrocomia* cultivation and breeding programs.

## Introduction

In temperate and strongly seasonal tropical regions, most plant species undergo a community-wide reproductive resting phase, driven by pronounced climatic constraints such as low winter temperatures or prolonged dry periods. These conditions result in a strict, synchronous annual flowering cycle for much of the plant community. In contrast, in tropical environments where seasonal climatic constraints are less restrictive, showing shorter or less severe dry periods or the absence thereof, species exhibit more diverse reproductive phenologies [1,2]. These range from unimodal, bimodal, to continuous flowering patterns occurring within annual, inter-annual, or multi-annual cycles [3–7]. The primary drivers of phenological patterns in tropical environments are precipitation, temperature, and light availability (intensity and exposure length), additionally influenced by local environmental conditions (e.g., soil quality, topography) ([8], and references therein).

The family of palms, Arecaceae, do not exhibit physiological dormancy since their stem ontogenesis and cells remain continuously active [9,10]. This largely explains their inability to tolerate cold temperatures, confining them primarily to tropical and subtropical regions of the world. Palms show a high sensitivity to seasonal climate. Environmental factors acting as triggers can either slow down or accelerate their growth and metabolic activity [10], as well as their reproductive life cycle. Palms exhibit a wide variety of complex mating strategies and a high diversity in phenological patterns across species, even within the same plant community [5]. Several studies have provided evidence that seasonal climatic variability can profoundly influence palm phenology, for instance in *E. guineensis* [11–13]; *Butia purpurascens* [14], *Bactris glaucescens* [15]; *Attalea phalerata* [15,16]; *Mauritiella armata* [17] and *Mauritia flexuosa* [18,19]. The potential role of photoperiodic cues, thus, cannot be entirely excluded, as flowering triggers remain elusive for many species [5,20–22]. Furthermore, adverse weather conditions, such as drought events, can strongly impact flowering and fruiting cycles, reducing sex ratios and fruit set in major palm crops like *E. guineensis* [12,23,24]*, Cocos nucifera* [25], and *Euterpe oleracea* [26]. In palms, each leaf axil bears a potential inflorescence bud [27,28], whose development from initiation to flowering can take several months to years, as seen in *E. guineensis* where the process takes 24–30 months [29]. This extended developmental timescale suggests that palm phenological timing is shaped by multiple environmental cues, but also increases vulnerability to adverse weather conditions. This has important implications not only for tree crop productivity but also for broader agricultural expansion strategies under unpredictable climate variability and extreme weather events [30,31].

In recent decades, interest in the economic valorization of the oilseed palm genus *Acrocomia*, in particular the species *Acrocomia aculeata*, *Acrocomia totai*, and *Acrocomia intumescens*, as an alternative source of vegetable oil to the African oil palm (*E. guineensis*) has increased significantly [32]. This growing interest has also prompted a focus on the reproductive biology of *Acrocomia*, primarily its inflorescence and fruit morphophysiology [30,33–37]. In Brazil, which hosts the highest diversity of wild *Acrocomia* populations [38,39], flowering typically occurs during the austral spring, spanning from August to February, with peak anthesis in November and December [36]. As *Acrocomia* flowering is generally observed to coincide with the onset of the rainy season in Brazil, precipitation has been proposed as a potential driver of its phenology [36]. Yet, there is evidence that it does not act as a proximate cue in the three months preceding flowering [33]. In addition, observations from other regions ([30,40] and references therein) cast doubt on the generality of the hypothesis of precipitation as cue for *Acrocomia*. For example, Carreño-Barrera et al. (2021) [30] reported for a Colombian region located North of the Equator, a flowering season from December to May, with a peak in March, during the region’s dry season. This observation leaves uncertainty about the climatic factors regulating flowering seasonality in *Acrocomia*. However, in each of these studies it was observed that flowering in *Acrocomia* was highly synchronous indicating a photoperiodic control [41,42], which has not been proven yet. Additionally, whether precipitation could acts as a long-term cue in *Acrocomia*, similar as in *M. flexuosa* [19], remains unclear.

This study therefore aimed to improve understanding of flowering periodicity, ranging from interannual changes to variability among genotypes, and the potential factors, namely temperature, precipitation, solar radiation, and photoperiod, influencing flowering phenology in *Acrocomia*, a field that has received little attention to date. We hypothesize that solar radiation and photoperiod, due to their regular and predictable nature, are the primary environmental cues regulating *Acrocomia* flowering seasonality.

Furthermore, understanding both within- and between-season fluctuations in *Acrocomia* flowering phenology is crucial for predicting fruit set and productivity, and for guiding conservation, cultivation, and expansion strategies. Within-season timing affects pollination success and fruit set [3,43], while between-season variation reflects long-term responses to climate [44].

Specifically, we address the following research questions:

1. How do flowering frequency and patterns vary over time and what temporal dynamics characterize flowering phenology in *Acrocomia*?
2. Which abiotic factors primarily drive the seasonal timing of flowering?
3. What are the broader implications of our findings for the strategic planning and management of *Acrocomia* production systems?

We assessed phenological traits of six *Acrocomia* accessions from geographically diverse regions in South America with a focus on Brazil, using circular statistics across three flowering seasons from 2019 to 2021. All accessions are grown in the Active Germplasm Bank of *Acrocomia* (BAG-Macaúba), located in the Mata Atlántica in south-eastern Minas Gerais, Brazil.

## Materials and methods

### Study species

*Acrocomia* species are endemic to the subhumid tropical regions of the Americas and demonstrate a high adaptability to diverse soil and climate conditions. *Acrocomia* occurs naturally in regions with annual mean temperatures ranging from 15°C to 35°C and annual rainfall between 1000 and 1900 mm [45], while regions with higher precipitation are less favorable. In areas with annual precipitation below 1000 mm, *Acrocomia* is primarily found in moist wetlands and along water bodies with well-draining soils [45–48]. Its natural occurrence is typically associated with tropical seasonally dry areas, such as regions classified according to Köppen-Geiger as having a tropical climate with a dry winter (Aw) and tropical monsoon (Af) [47,49]. *Acrocomia* can endure periods of water deficit and high temperatures [45,46,50,51].

The most widespread species is *A. aculeata*, which ranges from southeastern Brazil to southern Mexico, with major population centers in the Mata Atlântica and Cerrado regions. *A. totai* and *A. intumescens,* however, are distributed in distinct regions of South America. *A. totai* occurs primarily in the southwest of Brazil, Paraguay, Bolivia and Northern Argentina, especially within the Pantanal and Chaco biomes. In contrast, *A. intumescens* is restricted to the northeast of Brazil, where it is associated with the Caatinga biome and related ecotones.

**Fig 1:**
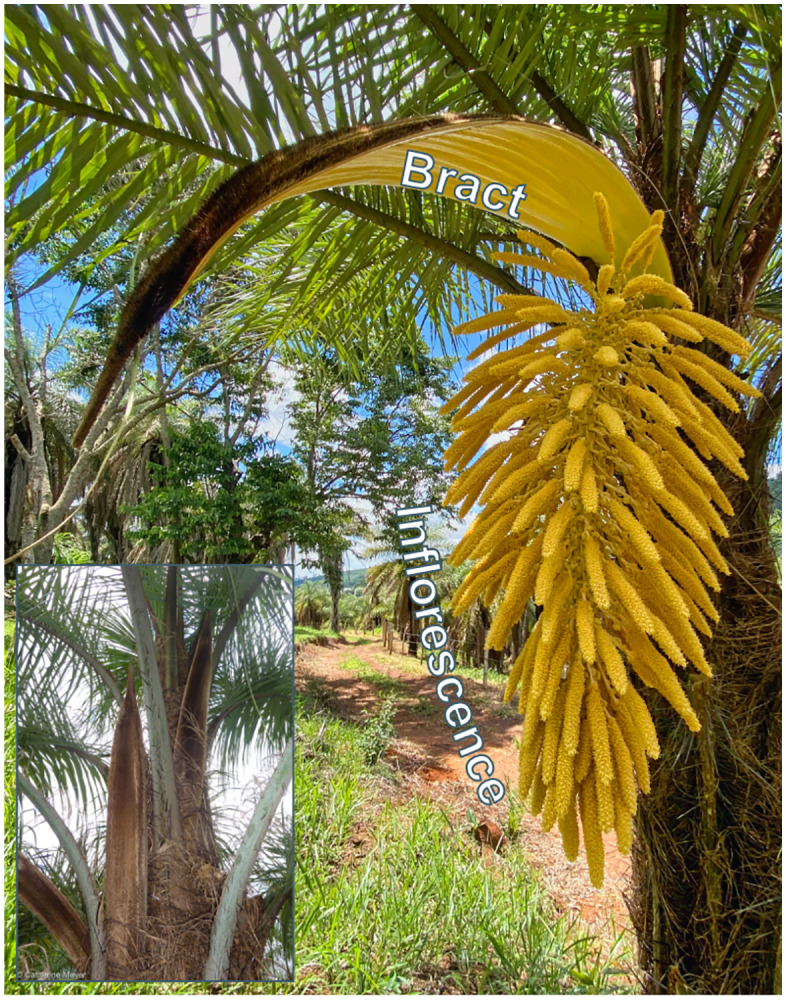
Inflorescence of *Acrocomia aculeata.* While emerging, the inflorescences are enclosed in a bract, which is woody and covered in brown trichomes on the outside with the occasional aculeus. The bract bursts at an abaxial, longitudinal slit and the inflorescence is released (= flowering event). At that point, pistillate flowers are already receptive, whereas staminate anthesis starts around 24 hours after bract opening. Small picture: Emerging, closed bracts in the palm crown. *A. aculeata* is here the representative of the studied species; bract-opening mechanics and overall inflorescence structure do not differ substantially from *A. intumescens* and *A. totai* [35].

*Acrocomia* is a monoecious and protogynous palm. Its large panicle inflorescences are protected by a peduncular and woody bract, which splits abaxially (Fig 1). The rachillae bear both staminate and pistillate flowers, with the pistillate flowers becoming receptive before the staminate flowers reach anthesis. This favors cross-pollination facilitated mainly by insect pollinators, including flower weevils (*Andranthobius* spp.; Derelomini) and small sap beetles (*Mystrops* sp.; Mystropini) [30,52]. Therefore, at least two inflorescences are necessary for a successful pollination event, one acting as pollen donor and the other as pollen recipient.

### Study site

The study was carried out at the Active Germplasm Bank of *Acrocomia* (BAG-Macaúba), an ex-situ collection of over 300 maternal families (Accessions) located at the experimental station of the Federal University of Viçosa in Araponga, Minas Gerais, in the South-East of Brazil (latitude 20°40′1′′S; longitude 42°31′15′′W; altitude of ~1000 m). The BAG-Macaúba is registered as an official repository by the Brazilian Board of Genetic Heritage (# 084/2013-SECEX/CEGEN). The climate is characterized as a humid highland climate with rainy, warm summers and dry winters and is classified under Köppen as Cwb [53]. The soil in the area is considered a red-yellow Latosol (FAO/WRB: Ferralsol) with a sandy-clay texture [54].

**Fig 2:**
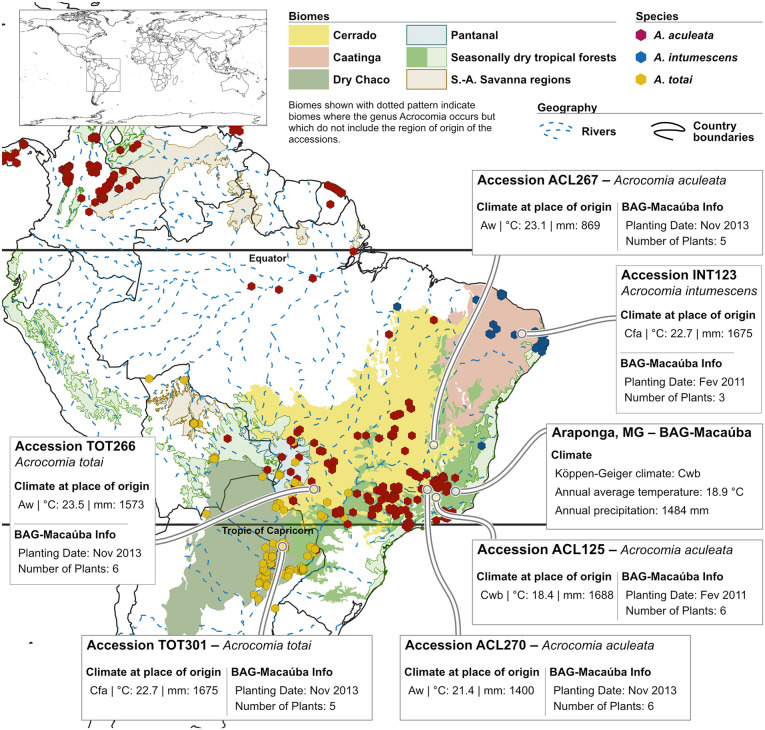
The regions of origin of the six *Acrocomia* accessions used in this study. The map shows the major South American biomes in which *Acrocomia* occurs [40,47,55,56]. The Köppen-Geiger climate classification, annual mean temperature and annual precipitation for each accession’s region of origin are provided. In addition, information is provided on the planting time in the BAG-Macaúba and the number of plants that were evaluated. The climate information and Köppen-Geiger climate classification per region are derived, respectively, from climate-data.org and koppen.earth (both accessed 24 Sept. 2025). Map created by the author in QGIS 3.40.0-Bratislava (QGIS Development Team, 2025). Ecoregion boundaries were obtained from Olson et al. (2001) [57] (WWF ecoregions dataset); river centerlines and physical and cultural boundaries were sourced from Natural Earth [58]. The distribution points are derived from GBIF.org (accessed 24. Sept. 2025), where only living specimens outside conservation sites (e.g., botanical gardens) were considered. All cartographic styling were performed by the author.

Six accessions (31 palms in total) were selected according to the criteria outlined in Meyer et al. (2024) [35] which included (1) confirmed flowering in 2018, (2) the number of palm trees available per accession, (3) the height of the palm trees for labor safety reasons, and (4) the geographic origin of each accession (Fig 2). The accessions were named based on the *Acrocomia* species they were allocated to, as a 3-letter prefix (INT = intumescens; ACL = aculeata; TOT = totai) followed by their accession number from the BAG-Macaúba database (Motoike S., unpublished data, 2018).

### Inclusivity in global research

The study was conducted under the framework of a Memorandum of Agreement between the University of Hohenheim (UHOH) and Federal University of Viçosa (UFV), signed in 2018. In addition, a Material Transfer Term was signed between UFV and UHOH on March 4^th^, 2020 for any exchange of plant, soil or other research material between the two institutions. Additional information regarding the ethical, cultural, and scientific considerations specific to inclusivity in global research related to this study is included in the Supporting Information (S7 Checklist).

### Monitoring of flowering events

In the context of this study, the term “flowering event” will hereafter be used to specifically refer to the event of bract opening for each individual inflorescence (Fig 1). This marks the start of flowering for the given inflorescence and serves as the key time point for tracking its reproductive development throughout the flowering season. The term “flowering onset” is defined as the first flowering event of each palm within each accession and season. The flowering events of the accessions were monitored from September to February over the years 2019, 2020, and 2021. These periods are referred to as FS2019, FS2020, and FS2021, collectively termed “flowering seasons” throughout the paper. In total, 382 inflorescences were recorded across all flowering seasons. Flowering was monitored two to three times per week under favorable weather conditions guaranteeing the accessibility to the plantation, and with at least one assessment conducted weekly under unfavorable weather conditions. Because not all flowering events could be dated precisely to the day, all observations were aggregated into half-month periods for analysis. Half-month refers to two periods of each month: the 1st to the 15th (1H) and the 16th to the end of the month (2H). In the following, each half-month will be abbreviated as 1H or 2H, followed by a three-letter month abbreviation, and if needed, the year (e.g., 2HDec2019 for the second half of December 2019)

### Circular statistics for flowering phenology

To assess the flowering pattern within and between seasons, we used circular statistics. Originally developed for data such as wind direction or animal migration patterns [59], the scope of circular statistics has since expanded to include phenological studies. In contrast to linear methods, which introduce discontinuities and artificial boundaries like the start and end of a year, circular statistics maintain the continuity of time, reducing bias and misinterpretation when dealing with circular data [1,59,60]. This is beneficial in tropical and subtropical ecosystems in particular of the Southern hemisphere, where phenophases often extend across the entire calendar year [1,61]. For circular statistics, time periods were converted into angles, with each day of the year (DOY) corresponding to approximately 1° and a half-month interval representing 15° on the circle.

For DOY (1–365) to angles (in degrees):


$$\begin{array}{c} {Angle}_{DOY}\;{(}^{\circ })=\;\frac{DOY}{\text{365}}\;\times {\text{360}}^{\circ } \end{array}$$ (1)

For half-month (1–24) to angles in degrees:


$$\begin{array}{c} {Angle}_{halfmonth}\;{(}^{\circ })=\;\frac{halfmonth}{\text{24}}\;\times {\text{360}}^{\circ } \end{array}$$ (2)

Using the “circular” package in R (Posit Software, Version 2024.09.0 + 375), we calculated the circular mean, circular median, angular deviation, and mean resultant length at the level of the flowering season. The mean resultant length (rho) is a measure for the concentration or dispersion of circular data, ranging from 0 (uniformly distributed) to 1 (perfectly clustered at a single angle).

The onset of flowering for each palm tree within an accession was defined as the date of its first flowering event. These individual onset dates were then used to calculate the circular mean and mean resultant length of flowering onset. For graphical presentation, the circular statistical measures were back transformed into half-month or DOY, where necessary.

To test for differences in flowering phenology, flowering dates were analyzed as angular data to capture their seasonal distribution among accessions within seasons and between seasons (FS2019, FS2020, FS2021). Two analyses were performed: one using all flowering events to calculate mean flowering dates, and another using only the first flowering event per palm (flowering onset). Data were grouped either by accession within each season (within season assessment) or by season within each accession (between season assessment). Differences in mean flowering direction were assessed using multivariate analysis of variance (MANOVA) on sine and cosine transformed angular flowering data (package npmv, R, Posit Software, Version 2024.09.0 + 375) [62]. Differences in angular dispersion among accessions were evaluated with the Wallraff test (package circular, R, Posit Software, Version 2024.09.0 + 375), which compares concentration of angles around the mean using the Kruskal-Wallis test (R Documentation, 2024).

### Climate and photoperiod data

**Fig 3:**
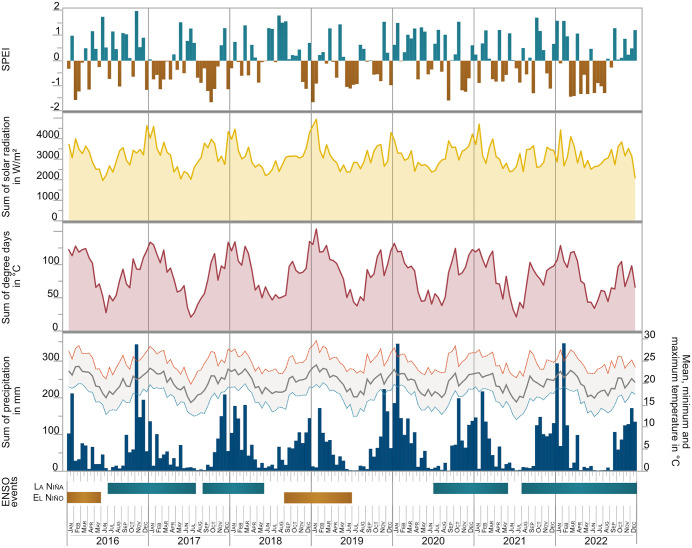
The climate variables in Araponga, Minas Gerais, Brazil from 2017 to 2022. They are shown in half-month aggregations from top to bottom: Standardized Precipitation-Evapotranspiration Index (SPEI), solar radiation, degree days, precipitation, mean temperature and minimum and maximum temperature. El Niño Southern Oscillation events (as defined by the NOAA Climate Prediction Center [66]) are shown as horizontal bars. El Niño is typically associated with drier conditions, while La Niña is associated with increased rainfall in south-eastern Brazil.

**Fig 4:**
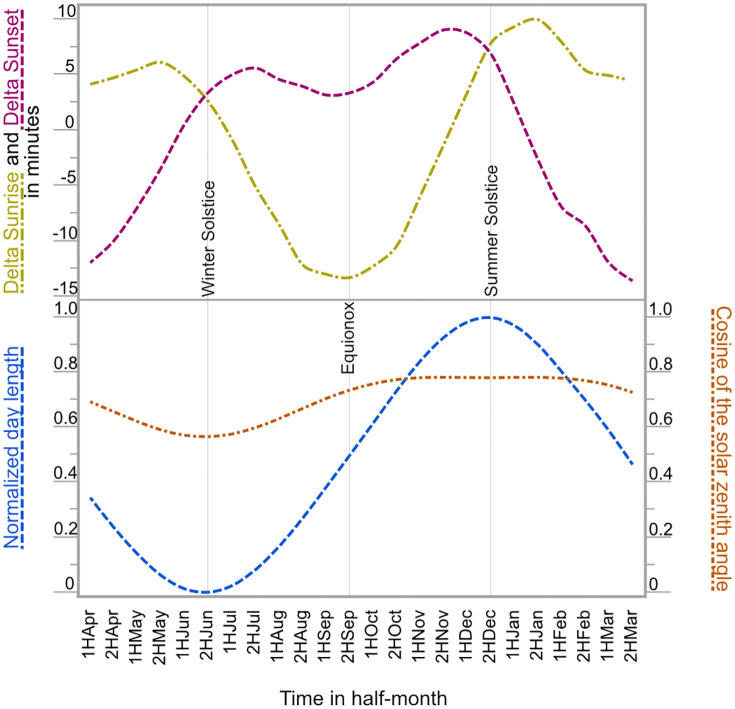
Photoperiod metrics for Araponga, MG, Brazil, calculated using NASA Goddard Institute for Space Studies data [65]. Top: difference between sunrise and sunset times over a half-month period. A negative sunrise/sunset delta means that the time of the event was earlier at the end of the period than at the beginning, and vice versa. A difference of 0 would indicate no change between the start and end of the period. The higher the absolute value, the more pronounced the difference and the greater the change. Bottom: Mean normalized day length and mean cosine of the solar zenith angle at solar noon.

The daily weather data were obtained from the AgEra5 dataset [63] through the Climate Engine platform [64] for the period of 01/01/2016 to 31/12/2022 (Fig 3). The photoperiod data were downloaded from the NASA Goddard Institute for Space Studies [65] (Fig 4).

From the full set of AgERA5 climate variables, we selected those that were known to influence palm phenology and were also considered relevant from ecological and agricultural perspectives. To avoid multicollinearity, we examined the Spearman’s rank correlation between all the candidate variables. The final selection included precipitation, solar radiation, degree days, and the Standardized Precipitation-Evapotranspiration Index (SPEI). Degree days were calculated from daily minimum and maximum temperatures in accordance with the methodology proposed by Suresh et al. (2021) [67]:


$$\begin{array}{c} Degree\;Days\;(C^{\circ })=\;\left(T_{min}-T_{b}\right)+\;\frac{\left(T_{max}-T_{min}\right)}{\text{2}} \end{array}$$ (3)

As the base temperature of *Acrocomia* is unknown, the base temperature of 15°C of the African oil palm (Suresh 2020) was used. As both palms share a similar temperature range, we considered it the closest alternative.

In line with Borchert et al.’s (2005, 2015) [41,68] recommendations for tropical and subtropical regions, we retained normalized day length, sunrise and sunset times, and the daily insolation-weighted cosine of the zenith angle as photoperiod variables. The zenith angle describes the position of the sun relative to the vertical. Incorporating its cosine, weighted by daily insolation, provides a more physiologically meaningful approximation of the radiation incident on the canopy surface, influencing light availability, photosynthesis and growth patterns [69]. The day length was normalized according to the following formula


$$\begin{array}{c} {day\;length}_{Normalized\;}=\;\frac{day\;length-\;{day\;length}_{short,year}}{{day\;length}_{long,year}-\;{day\;length}_{short,year}} \end{array}$$ (4)

where day length is the number of daylight hours on a given day, day length_short, year_ and day length_long, year_ are, respectively, the daylight hours of the shortest and longest days of the year.

All the selected climate and photoperiod factors were then aggregated to the half-month as summarized in Table 1, to align with the time scale of the flowering phenology dataset. The SPEI was calculated using the SPEI R package (Posit Software, version 2024.09.0 + 375) to identify periods of drought and wetness based on the aggregated precipitation and Hargreaves–Samani potential evapotranspiration.

**Table 1:** An overview of the aggregation methods of all environmental factors used.

Variable	Unit	Aggregation
Precipitation	mm	Sum
SPEI	dimensionless	calculated
Solar radiation	W/m2	Sum
Degree days	°C	Sum
Normalized day length	dimensionless	Mean
Delta sunrise	minutes	Difference between end and start of half-month, based on Borchert et al. (2005) [70]
Delta sunset	minutes	Difference between end and start of half-month, based on Borchert et al. (2005) [70]
Sunlight-weighted cosine of the Zenith Angle	dimensionless	Mean

We calculated the two-week change in sunrise and sunset times (delta sunrise and delta sunset) as the difference between their times at the start and end of each half-month period [41].

### Seasonal decomposition of climate factors

**Fig 5:**
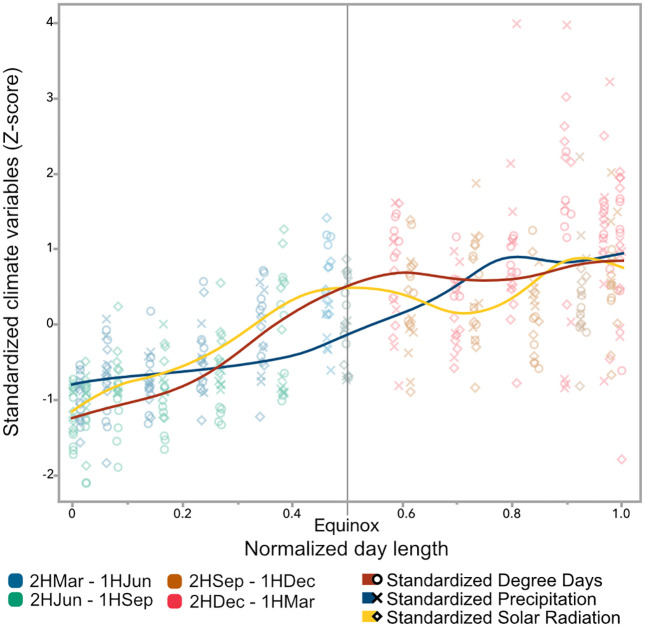
Half-month aggregated climatic factors, standardized using z-scores, plotted against normalized day length. The mean values of the standardized factors are shown alongside the half-month data points. The equinoxes are marked at a normalized day length of 0.5. The dataset, which covers the period from January 2016 to December 2022, was derived from AgERA5 [63,64]. Normalized day length was calculated using NASA Goddard Institute for Space Studies data [65].

Exploration of the climate data showed a hierarchical clustering of the climate data into winter (April to September) and summer periods (October to March) (Fig 5). Therefore, we performed a seasonal decomposition of time series using LOESS in R (Posit Software, Version 2024.09.0 + 375) on the 168 half-month periods (1HJan2016–2HDec2022). To minimize border effects in the seasonal decomposition model, one year of data preceding and following the period of interest of 2017–2021 was included, ensuring a more stable estimation of the seasonal decomposition components within the focal period (S1 Fig). Seasonal decomposition separates a time series into three components: the trend, representing long-term changes, the seasonal component, capturing regular repeating patterns, and the residuals, containing short-term irregular deviations not explained by trend or seasonality. These residual deviations are ecologically relevant because anomalies in temperature, precipitation, or drought (SPEI) can disrupt the timing (delay or advance) and magnitude of phenological events depending on species and phenophase, acting as environmental triggers [71,72]. For our analysis, we retained only the trend and residual components, excluding the seasonal signal, to focus on both long-term patterns and potential impacts of short-term anomalies on phenology.

### Moving window correlation analysis

We conducted a moving window correlation analysis using Spearman’s rank correlation to assess the relationship between the peak flowering period and aggregated environmental factors, as well as trends and residual components extracted from seasonal decomposition. As for different palm species, the impact of environmental factors was found to be up to two years prior to the phenophase [23,28,29], we extended the analysis up to two and a half years before the flowering peak and performed it separately for each season and factor. The flowering event window was fixed at five half-months, spanning from 2HOct to 2HDec for each season, where the average half-month of the seasons’ peak was the middle (2HNov). The environmental factor window, also five half-months long, was shifted back by an increment of one half-month for a total of 60 half-months. Before analysis, inflorescence counts were log10-transformed to standardize flowering intensity across seasons, highlighting relative temporal patterns. Raw climate data (precipitation, degree days, solar radiation) were z-score standardized, while trend and residual components from seasonal decomposition were used unstandardized. To assess the significance of Spearman’s rank correlation coefficients, confidence intervals were calculated using the method described by Bonett and Wright (2000) [73]. All analyses, including the generation of heatmaps, were performed in R (Posit Software, Version 2024.09.0 + 375).

## Results

The BAG-Macaúba at Araponga, Minas Gerais, Brazil provides a unique opportunity to study the phenology of accessions originating from populations adapted to diverse climatic and photoperiod regimes, under a common garden setting.

**Fig 6:**
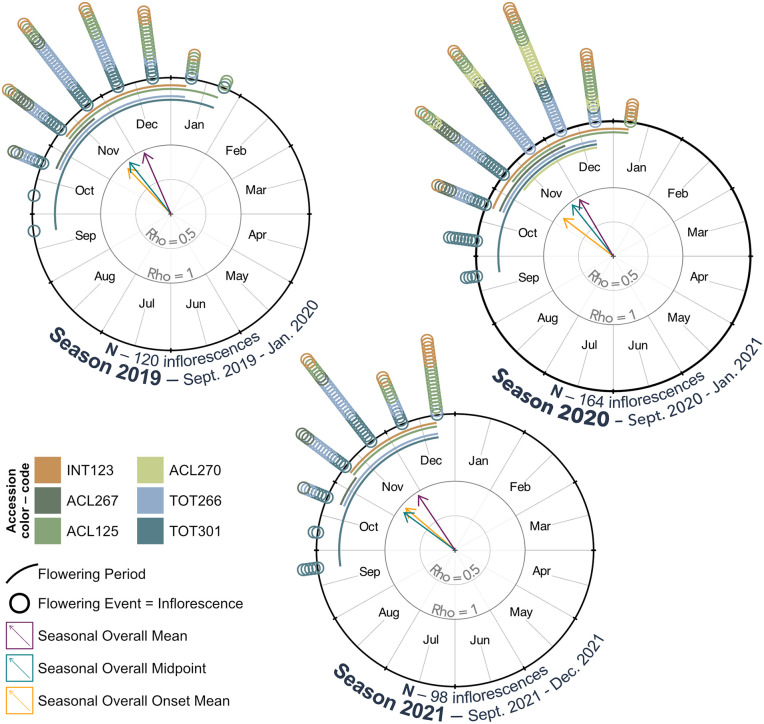
Circular flowering pattern across FS2019, FS2020, and FS2021 of six accessions in Araponga, MG. **Brazil.** The seasonal overall mean, midpoint, and onset mean are circular measures, with the length of the arrows representing the mean resultant length (rho). The colored lines represent the flowering range for each accession, and the small circles indicate the number of flowering events per half-month.

*Acrocomia* flowering showed distinct temporal dynamics within and across years, with clear seasonality from September to January and peaks in late November (FS2019, FS2021) or early December (FS2020). Flowering onset was consistent across years, beginning in early September (DOY 320–329), around the spring equinox (Fig 6), coinciding with first rainy season rainfall, though inflorescence emergence occurred as early as mid-August. For each flowering season, MANOVA results showed no significant differences in mean flowering time among accessions (Table 2), implying that accessions tend to flower around the same mean date. However, the Wallraff test indicated that accessions differed significantly in their angular distribution around the seasonal mean within each season (Table 2), reflecting differences in the duration of flowering periods among accessions.

**Table 2:** Season-specific differences in flowering synchrony among accessions in Araponga (MG, Brazil). Statistical differences among accessions were tested separately for each flowering season (FS) using a MANOVA on sine- and cosine-transformed angular data, as well as the Wallraff test, which is a circular statistical method that assesses whether multiple groups differ significantly in their angular dispersion. The mean resultant length ‘Rho’ is a measure of the clustering strength of flowering events in Araponga, MG, Brazil, around the mean date, where stronger synchrony is indicated by values that are closer to 1. P-values indicating significant differences among accessions (a < 0.05) are shown in bold.

				MANOVA				Wallraff test Statistics		
	Season	Mean DOY	Rho	(ANOVA-type statistic)	df1	df2	Permutated p-value	chi-squared	df	p-value
Flowering Onset	FS2019	328.5	0.93	3.821	4	35.06	0.525	6.159	4	0.188
	FS2020	322.0	0.91	0.216	5	49.54	0.99	6.589	5	0.253
	FS2021	319.3	0.92	0.512	4	30.26	0.91	7.834	4	0.098
Seasonal Flowering Mean	FS2019	341.6	0.97	1.724	4	235	0.619	14.05	4	**0.007**
	FS2020	334.2	0.97	4.535	5	298	0.062	20.31	5	**0.001**
	FS2021	331.2	0.95	1.433	4	131	0.643	12.29	4	**0.015**

Seasonal flowering patterns in Araponga show clear seasonality, as indicated by the overall means (DOY 332–342) and midpoints (DOY 321–333) of flowering time (Fig 6 and Table 3). Statistical analyses (MANOVA on sine and cosine-transformed angular data, and Wallraff tests) showed that, for most accessions, flowering patterns remained consistent across years (Table 3). This means that the timing and distribution of flowering did not significantly vary between seasons for the majority of accessions. Only two accessions, ACL270 and TOT301, showed differences in their flowering pattern.

**Table 3:** Seasonal mean flowering dates and synchrony indices of accessions in Araponga (MG, Brazil). Seasonal mean flowering dates are presented as circular means transformed to linear “day of the year” (DOY). The mean resultant length (ρ) quantifies the degree of clustering of flowering events around the mean date, with values closer to 1 indicating stronger flowering synchrony. Statistical differences among seasons within each accession were tested using a MANOVA on sine- and cosine-transformed angular data and the Wallraff test, which assesses differences in angular dispersion among multiple groups. Accession ACL270 produced no inflorescences in FS2019 and FS2021. P-values indicating significant differences (α = 0.05) are shown in bold.

				MANOVA				Wallraff test Statistics		
Accession	Season	Mean (DOY)	Rho	(ANOVA-type statistic)	df1	df2	Permutated p-value	chi-squared	df	p-value
INT123	FS2019	352	0.952	3.80	2	38.0	0.167	0.38	2	0.826
	FS2020	355	0.931							
	FS2021	355	0.957							
ACL125	FS2019	361	0.953	0.311	2	76.9	0.825	0.79	2	0.673
	FS2020	352	0.956							
	FS2021	355	0.956							
ACL267	FS2019	321	0.985	0.363	2	51.3	0.833	0.06	2	0.973
	FS2020	321	0.975							
	FS2021	317	0.995							
ACL270	FS2019	na								
	FS2020	345	0.977							
	FS2021	na								
TOT266	FS2019	337	0.957	3.540	2	184.0	0.155	0.28	2	0.870
	FS2020	335	0.955							
	FS2021	326	0.969							
TOT301	FS2019	337	0.902	9.287	2	204.4	**0.007**	0.42	2	0.812
	FS2020	315	0.914							
	FS2021	315	0.916							

Interannual variation reflected changes in flowering event number and range; for example, accession ACL270 flowered only in FS2020, while ACL267, TOT266, and TOT301 flowered earlier on average than ACL125 and INT123 (Fig 6). *A. totai* accessions flowered earlier and over a wider range than *A. aculeata*, with consistent differences also observed among accessions of the same species and individual trees of the same accession (S2 Fig).

Flowering coincided with early summer, following the spring equinox and peaking before the summer solstice (S3 Fig), corresponding to a climatic transition from dry, cool winter to warmer, wetter conditions. The flowering season is defined as the period where flowering events occur, so as the activity of bract opening. However, inflorescences require several weeks from bract emergence to opening (Meyer, pers. obs.), so earlier environmental cues likely influence flowering phenology.

**Fig 7:**
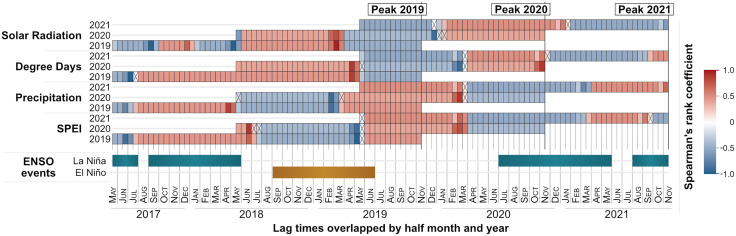
Spearman’s rank correlations between flowering peaks and seasonal climatic trends in Araponga (MG, Brazil). Spearman’s rank correlation coefficients between flowering peaks (FS2019, FS2020, and FS2021) and climatic trends obtained from seasonal decomposition, calculated for half-monthly intervals ordered by season. Crossed-out coefficients are not significant based on their confidence intervals. The indicated half-month corresponds to the midpoint of the moving correlation window.

Seasonal decomposition revealed that flowering peaks were negatively associated with long-term decreases of climate variables and positively associated with their increases (Fig 7). However, the direction of these long-term climate trends differed in the months preceding each flowering season, spanning both dry and rainy periods, and thus varied between years. The El Niño Southern Oscillation seem to have a certain impact on the long-term climate trend, though not consistent. In the twelve half-months preceding FS2019, precipitation and SPEI increased, while degree days and solar radiation declined (Fig 7), resulting in positive correlations with the former and negative with the latter. In contrast, during FS2020, when the climate trend shifted around the fall equinox toward a long-term trend of rising degree days and solar radiation and declining precipitation and SPEI, the correlations reversed.

**Fig 8:**
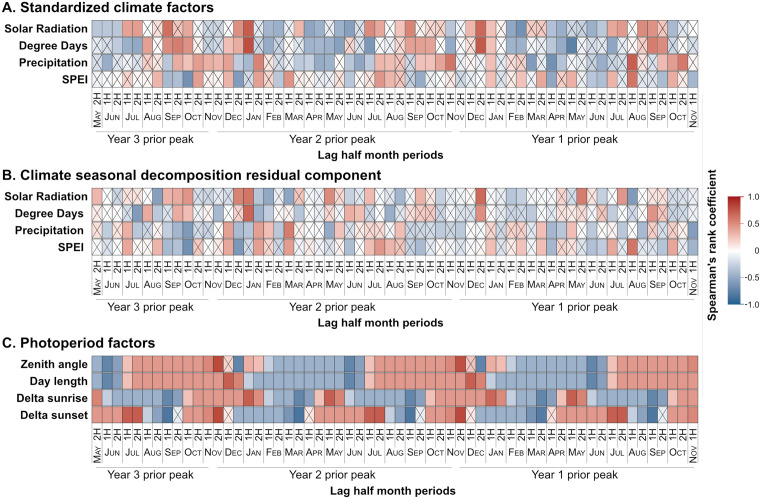
Mean Spearman’s rank correlations between flowering peaks and photoperiodic or climatic factors across lags in Araponga (MG, Brazil). Mean Spearman’s rank correlation coefficients between flowering peaks and (A) photoperiodic factors, (B) standardized climatic factors, and (C) residuals component of the seasonal decomposition, calculated for successive lags. The indicated half-month corresponds to the midpoint of the moving correlation window. Periods with non-significant correlations, based on confidence intervals, are crossed out. Detailed results for each lag and season are provided in S4 Fig.

Flowering peaks also correlated positively with increasing photoperiod and negatively with decreasing photoperiod, including subtle shifts in sunrise and sunset (Fig 8A). However, no consistent differences in photoperiod–flowering relationships were observed between flowering seasons, as the temporal structure of photoperiod remains constant across years, resulting in similar lagged correlation patterns aligned to the same dates each season (S4 Fig).

Across various lag times, seasonal mean correlations between flowering peaks and standardized climate variables were mostly weak or non-significant (Fig 8B). To identify potential trigger periods, we took the mean of the correlation coefficients across all three seasons for each lag, aiming to identify lags to find consistently directed associations. Most lag periods showed minimal association, suggesting limited direct climate influence or opposing effects on the flowering seasons.

Stronger positive correlations with degree days and solar radiation were observed at lag times around the summer solstice one (2HDec) and two (1HJan) years prior to flowering, periods when these variables peak annually.

Precipitation and SPEI generally showed weak associations with the flowering peak, except in 1HAug, which exhibited a stronger positive correlation. August marks the peak of the dry season, when precipitation reaches its annual minimum. Nevertheless, short, intense rainfall events, such as thunderstorms, occur regularly in the mountainous region of Araponga. SPEI indicated that no winter between 2016 and 2021 was severely or extremely dry; notably, the winter of 2018 was very wet (SPEI 1HAug2018: + 1.8). Despite these conditions, the residual component of the seasonal decomposition, representing irregular deviations in climate, showed no clear relationship with flowering patterns, suggesting that short-term climatic anomalies did not strongly influence the timing or distribution of flowering (Fig 8C).

A closer examination of Spearman’s rank correlation coefficients by lag and season (S4 Fig) reveals that correlations between precipitation and SPEI (and their residual component) and the timing of the flowering peak are strongly positive at a lag of 24 half-months. The slight shift in correlation timing— late January of 2017 (2HJan2017) and 2019 (2HJan2019) for FS2019/FS2021 and early February 2018 for FS2020—may reflect differences in when developmental triggers occurred, potentially being linked to the observed offset in flowering peaks (2HNov in FS2019 and FS2021; 1HDec in FS2020). This suggests that rainfall and drought anomalies during this period influence the onset or progression of reproductive development that determines when flowering occurs. January to February generally marks a drier interval within the rainy summer in Araponga (Fig 3). Although the precipitation and SPEI residual components show no consistent magnitude during these months across years, their long-term temporal alignment with flowering peaks indicates that these associations are confined to discrete, recurrent periods rather than reflecting consistent effects of overall water availability.

## Discussion

Our results show that photoperiod is a key environmental cue for flowering in *Acrocomia* in Araponga, MG. The pronounced and recurrent flowering seasonality, together with low inter-annual variation among accessions despite climatic fluctuations, suggests that photoperiod acts as the dominant anticipatory signal regulating seasonality under the conditions in Araponga during the study period, while episodic climatic variation fine-tunes the timing of flowering events.

In Araponga, flowering of *Acrocomia* typically starts around the spring equinox, peaking before the summer solstice, characterized by minimal interannual variability (Fig 6). *Acrocomia* exhibits high regional synchrony of flowering during periods of increasing daylength, with a peak around the summer solstice, as reported across the species’ range from southwest to central Brazil [33,74,75]. Carreño-Barrera et al. (2021) [30] reported a flowering peak in March and a flowering season from December to April after the rainy season in northern equatorial Colombia. In the Mexican State of Veracruz, one of the northernmost regions of occurrence of *Acrocomia* [47], flowering extends from March to September, spanning the period between the spring and fall equinox, and broadly coinciding with the rainy season [76]. Though flowering onset at the dry–rainy season transition is reported in Brazil [33,74,75], comparison of observations from Brazil to Colombia and Mexico indicates a more consistent alignment with photoperiod (day length, sunrise or sunset timing, or related variables) from shortly after the winter solstice through the summer solstice and slightly beyond, whereas associations with climatic seasonality are less consistent across regions. This pattern is consistent with the observations that increases in photoperiod and daily insolation are associated with tropical plant phenology irrespective of the hemisphere [68,77,78], resulting in latitude specific seasonal flowering peaks [41], which was hypothesized for *E. guineensis* [12]. *Acrocomia* is a genus typical of the Brazilian Cerrado biome [79] and shares phenological patterns with other woody species in the Cerrado, often flowering at the transition from the dry to rainy season [80,81]. Rocha et al. (2024) [81] suggest that this seasonality is shaped by the dominant macroclimate of the region, influenced by photoperiod, while microclimate and soils modulate interannual variation. The synchronization of flowering within predictable periods likely aligns reproduction with favorable abiotic conditions and with peak pollinator activity, thereby promoting the ecological interaction necessary for successful reproduction, as has been commonly reported for tropical plants [44,98–100]. In this context, photoperiod provides a stable seasonal signal that may facilitate reproductive synchronization across years. Evidence from phylogenetically distant palm species supports the hypothesis that reproductive timing has evolved in synchrony with pollinator activity patterns, which in turn are shaped by abiotic cues [5,82,100]. Seasonal variation in daylength, sunrise and sunset times, and zenith angle may therefore function as a reliable signal of upcoming favorable conditions, facilitating coordinated reproductive effort across genetically diverse *Acrocomia* populations.

### Limited short- and long-term climate effects

While the pronounced flowering seasonality links to photoperiod, climatic factors likely shape fine-scale variation in flowering timing via longer-term developmental processes, while short-term climate effects take a minimal role.

Precipitation events in August prior to flowering showed a positive correlation with flowering peaks. This suggests a potential role of precipitation in modulating the timing of bract opening within a photoperiodically-defined flowering window, as the first bract-enclosed inflorescences were observed as early as mid-August. However, this signal was not consistently detected across the broader period preceding flowering onset and up to the flowering peak, where associations with climate factors were generally weak and variable between years. These findings show only partial agreement with the results reported by Lorenzi (2006) [33], who found no correlation between temperature, precipitation, and flowering timing in the three months preceding flowering in *Acrocomia* populations in Paraná. Despite the evident similarity between the climatic (i.e., periods of dry winter and rainy summer) and the flowering seasons (peaking in November) in Paraná and in Minas Gerais, it is plausible that the discrepancy between the studies can be attributed to the methodological differences in the length of the chosen observation windows (three months in Lorenzi (2006) [33], and half a month in this study). Whilst certain studies have found no link between flowering and preceding temperature or precipitation in a range of palm species [5,33,82], others have identified such effects in other palm species including *Mauritiella armata* [17], *Mauritia flexuosa* [18,19], *Attalea phalerata* [15,16], *A. funifera* [83], *Bactris glaucescens* [15], *Butia purpurascens* [14], *Cocos nucifera* [84], and *Sygarus romanzoffiana* [85]. Overall, this evidence suggests that responses to short-term climatic variation are species-specific and even differ between members of the same genus [5,82]. In Araponga, rainfall during the dry winter occurs mainly as convective thunderstorms, and the observed signal in August likely reflects these short moisture pulses, which provide temporary relief from drought stress. In seasonally dry ecosystems, brief rainfall events have been shown to relieve water deficit and stimulate photosynthesis, growth, and reproduction [86,87], potentially influencing pre-floral development and fine-tuning the timing of flowering events [86,88]. The inconsistency in the link between flowering timing and short-term precipitation suggests that water availability likely plays only a minor role in modulating flowering seasonality of the *Acrocomia* accessions in Araponga.

While precipitation does not play a major role in the timing of the flowerings season window, correlations with precipitation and SPEI, and their deseasonalized anomalies, one to two years before flowering indicate that climatic variability is potentially linked to processes occurring during reproductive development. However, these correlations are not accompanied by consistent patterns in the magnitude or direction of the climatic variables across flowering seasons, indicating that the reason for these associations is not straightforward and remains unclear. Despite the lack of consistency, strong positive correlations with rainfall and drought anomalies 24 half-months prior to the flowering peak (S4 Fig) suggest that short moisture deviations may affect early developmental stages of leaves or inflorescences in the apical meristem. In pleonanthic species of the Arecaceae, each leaf has the capacity to develop an axillary inflorescence bud [28]. Thus, the number of inflorescence buds is associated with the number of the subtending leaves, which is further influenced by resource availability [12,28,29]. Consequently, the leaf emergence rhythm dictates the timing of inflorescence formation. In species such as *Elaeis guineensis*, inflorescence initiation occurs approximately 24–30 months prior to anthesis, followed by a prolonged phase of differentiation and expansion before flowering [12,29,89,90]. Similarly, extended developmental timelines exceeding two years have been described for *C. nucifera* [91], which, like *E. guineensis*, undergoes a sequence of several growth phases of reproductive development at different growth rates [29,91]. It remains unclear whether the strong positive correlation with water availability 22 months prior to the peak primarily corresponds to the timing of inflorescence initiation, leaf primordia formation that will subtend future inflorescences, or both, as the developmental timeline of *Acrocomia* is currently unknown. Despite this uncertainty, the timing and consistency of these long-term climatic associations with the flowering peak support a multi-year developmental process from floral initiation to bract opening in *Acrocomia*.

The marked seasonality in precipitation, temperature, and solar radiation (Fig 3) in Araponga, regulates the vegetative and reproductive development of the *Acrocomia* accessions in the BAG-Macaúba. Independent of the accession, leaves that expanded from around the summer solstice until shortly after the fall equinox produced inflorescences (S5 and S6 Figs), coinciding with periods of abundant water, radiation, and temperature. The process of leaf emergence was suppressed during the late winter months and resumed with increasing rainfall and radiation around the spring equinox (see S3 Fig). Beside the leaf emergence rhythm, the flowering periodicity in pleonanthic Arecaeae depends on two additional processes: the initiation and survival of inflorescence buds and units, and their expansion leading to bract opening and pollen release [28]. Resource availability, metabolic activity, sugar levels in the stem, and hormonal signal have been identified as contributing factors to inflorescence development in Arecaceae [12,90,92–95]. Explicitly, findings in *E. guineensis* suggest that the limited availability of resources, particularly water and radiation, may regulate the development and abortion of inflorescences (9–11 months before harvest) by affecting the carbohydrate status of plants and resulting in source-sink imbalances [11,12,24,89]. The absence of inflorescences in leaves expanding in winter and spring suggests abortion under conditions of resource stress. Inflorescences did not always flower in strict sequence with leaf age (S6 Fig), further indicating delays in pre-floral development possibly linked to resource availability. Thus, resource limitations may cause inflorescence abortion or slow expansion at various crucial developmental stages in *Acrocomia*. Primordia dissection studies [29] would be required to verify these assumptions.

### Accession-specific variation in flowering event distribution

Despite the considerable geographic distances between their origins, the accessions flowered within the same seasonal window, suggesting that support for a single climatic factor acting as a universal trigger is not evident. There were no interannual differences in mean flowering time; however, accessions differed in their flowering period range, likely due to staggered flowering onset within accessions, the number of inflorescences produced per plant (more inflorescences resulting in a longer flowering period), and the flowering event rate (time between flowering events). Interannual variability was only evident in two accessions: ACL270, which flowered only in 2020; and TOT301, which showed a delayed onset and less clustered flowering events in 2019. At the start of this study, the plants were 6 and 8 years old, respectively, and still showed irregular reproductive behavior. We observed subtle but consistent differences in flowering onset, mean, and range among the various accessions (Fig. 6), as well as at the level of individual palms (see S2 Fig). Overall, this indicates that flowering seasonality is strongly synchronized and most likely driven by photoperiod, while accession-specific differences are mainly expressed in flowering intensity and event distribution within the season rather than in flowering timing within the year. While this distribution could in principle be influenced by climatic conditions through effects on short developmental phases such as inflorescence expansion or bract opening, any such effects would likely operate at a finer temporal scale (i.e., days) than the two-week resolution of this dataset. In addition, accessions may differ in their sensitivity to seasonal changes in light cues, including day length, sunrise or sunset timing, and solar zenith angle, which could contribute to the observed variation in flowering dynamics within the seasonal window. Such differences may reflect genetic adaptation to regional seasonal regimes [96,97], although the extent to which they represent genotype × environment interactions cannot be resolved within the single-site design of this study.

A number of limitations should be considered when extrapolating the discussed results beyond the study system. Firstly, the short study period of two to three flowering seasons captured only part of the interannual variability in flowering intensity, which was insufficient for some accessions. Secondly, while the inclusion of multiple genotypes from different origins within a common environment is a key strength, allowing partial separation of genetic and environmental effects, the absence of reciprocal trials across multiple sites limits the ability to assess the consistency of genotype-specific responses under contrasting conditions. Furthermore, as all accessions originated between 7° S and 25° S due to the availability of material in the BAG-Macaúba, including material from a broader latitudinal range, spanning both hemispheres, would strengthen the evaluation of how phenological patterns vary across environments and accessions. These constraints highlight the need for longer-term, multi-site experiments incorporating accessions across the species’ full distributional range. Such approaches would enable more robust testing of phenological patterns across environments and help resolve their drivers in *Acrocomia* beyond a correlational statistic, particularly when coupled with efforts to elucidate the underlying developmental mechanisms.

### Implications for cultivation and breeding

Flowering periodicity is a central component of *Acrocomia*’s reproductive biology and has direct implications for its commercial cultivation as a palm crop for vegetable oil. Understanding the species’ flowering phenology is critical for plantation design, as self-pollination often leads to fruit abortion due to inbreeding depression [30,101,102]. Sequential flowering within individual palms reduces the likelihood of self-pollination, while synchrony among palms in a population and continuous flowering throughout the season enhance cross-pollination [36]. This combination of traits promotes gene flow among genetically diverse wild plants and reduces the risk of reproductive failure [36,47].

Meyer et al. (2024) [35] reported substantial phenotypic variation among *Acrocomia* genotypes, with inflorescence traits differing across clones and species, highlighting opportunities to exploit this diversity to improve plantation resilience and yield stability. Effective plantation management must therefore consider *Acrocomia*’s reproductive biology to maximize reproductive success. Based on our results and prior studies, and the limitations outlined above, we provide several considerations for the design and establishment of commercial plantations.

1. Planting only closely related or genetically uniform material (e.g., clones) may lead to poor fruit set and yield instability. However, planting genetically diverse material—particularly important when using clones—can mitigate the effects of dichogamy and ensure effective cross-pollination, since selfing rarely results in fruit set [30,38,102]. The deliberate use of phenological diversity, as noted by Scariot et al. (1991) [36], enhances mating opportunities and supports reproductive success. We suggest planting genetically diverse material and, in the case of clones, using several clones originating from different regions. As hybridization between species is observed [39,103,104], they can act as pollen donor to each other. So, a mixture of clones of *A. totai*, A. *intumescens*, and A. *aculeata*, which all show diversity in inflorescence and fruit traits [35] and phenological patterns, may be particularly valuable.
2. As photoperiod appears to be the key environmental cue of flowering in *Acrocomia*, with climate factors playing a secondary role, integrating genotypes from different geographic origins or *Acrocomia* species into plantations is feasible. Flowering periods will seasonally align despite local adaptations, allowing use of diverse genetic material without disrupting flowering synchrony. From a breeding perspective, these findings imply a high degree of transferability of flowering seasonality across latitudes, while still allowing for selection on traits influencing flowering duration, flowering intensity and synchrony, which may be relevant for yield distribution and harvest management.
3. Integrating genotypes with earlier or later flowering windows, as well as narrower and wider flowering windows within the same plantation, has the potential to extend the flowering and harvest periods. On the one hand, this permits adaptation to specific production systems, ranging from monocropping to silvopastoral and silvoarable systems, to complex multistrata agroforestry, while ensuring sufficient overlap for cross-pollination. For instance, introducing various *Acrocomia* genotypes into silvopastoral systems could lead to staggered harvest windows, where livestock can be kept on site while being kept apart from active harvesting areas. Conversely, staggering harvests, which are the most labor-intensive periods, can enhance predictability and flexibility in labor and post-harvest management.

Alternating production years, a common feature in *Acrocomia*, should also be considered. Year-to-year fluctuations in inflorescence production (Fig 6) are influenced by plant carbon (C) reserves, which depend on photosynthetic activity [105,106]. Long-term climatic patterns affect resource accumulation and allocation, ultimately determining the number of inflorescences produced [13,90,107,108]. However, the causes and magnitude of alternating production years in *Acrocomia* remain unclear due to the lack of long-term data on yield formation and C reserves.

## Conclusion and outlook

This study is the first to examine the long-term influence of photoperiod and climatic factors on the flowering phenology of diverse *Acrocomia* accessions from across South America, grown under identical conditions. We show that *Acrocomia* exhibits highly synchronized flowering across years, reflecting a complex interplay of environmental cues. Photoperiod emerges as the consistently dominant factor, likely reflecting co-evolution with pollinator activity and enhancing reproductive success. This provides partial support for our hypothesis that solar radiation and photoperiod, due to their regular and predictable nature, would act as primary environmental cues regulating flowering seasonality. Solar radiation, though, showed highly inconsistent relationships with flowering timing. While precipitation contributes to phenological variation, its interannual inconsistency suggests that the traditional hypothesis of flowering being directly triggered by the onset of the rainy season is not supported.

Monitoring flowering phenophases in conservation plantations can identify accessions with similar or contrasting phenological traits, providing a practical tool for selecting planting material for commercial plantations. Maintaining clones with diverse inflorescence traits and phenological patterns promotes successful pollination and sustained productivity, buffering plantations against adverse climatic conditions that affect pollinators or pollen dispersal.

Current phenology studies, including ours, typically span only two to three flowering seasons and may not capture the full phenological cycle. We therefore recommend long-term monitoring of flowering and fruiting, particularly in conservation collections, combined with genotypic exchange among collections across South America to evaluate how identical genotypes respond to different environments. A detailed understanding of phenophase dynamics is essential for predicting production cycles, guiding genotype selection, and assessing vulnerability to climate change, all critical for realizing *Acrocomia*’s potential in food and biofuel industries.

Finally, advancing our understanding of flowering phenology and fluctuations in floral intensity requires studies on leaf and inflorescence initiation through primordia dissection; investigation of gene families regulating flowering; exploration of environmental, hormonal, and stress-response pathways affecting these genes; and integration of metabolic, genetic, physiological, and ecological perspectives through interdisciplinary approaches.

## Supporting information

S1 Fig Results of the seasonal decomposition of time series using LOESS on the 168 half-month period (1HJan2016–2HDec2022). (TIF)

S2 Fig Flowering event frequency per half-month for the 31 palms assessed during the 2019, 2020, and 2021 flowering seasons. (TIF)

S3 Fig Seasonal course of flowering activity in relation to photoperiod and climate factors. The x-axis represents half-month periods within a single year, beginning after the fall equinox in 2HMar. The panels show photoperiod-related variables, and mean values of standardized degree days, standardized precipitation, and SPEI for Araponga, MG, Brazil, averaged per half-month across 2017–2022. For each year, climate data were previously aggregated following the methodology described in the Material and Methods section. Log-transformed counts of flowering events are plotted on the same time scale to illustrate seasonal dynamics in relation to climatic and photoperiodic variation.
(TIF)

S4 Fig Spearman’s rank correlation coefficient for the flowering peak in relation to photoperiodic factors (A), standardized climate factors (B), and climate residual components (C), ordered by lag separately for the flowering season FS2019, FS2020 and FS2021. The indicated half-month corresponds to the middle of the moving correlation window. Periods showing non-significant correlations, based on the confidence intervals are crossed out.
(TIF)

S5 Fig Relationship between leaf age and bract opening of the corresponding inflorescence. Inflorescences do not necessarily open in relation to the age of their associated leaf. Data were collected during the FS2019 in Araponga, MG, Brazil.
(TIF)

S6 Fig Relationship between the half-month period of full leaf expansion and the bract opening of the corresponding inflorescence. Most inflorescences formed in the axils of leaves that expanded around the spring equinox preceding the flowering season. The time interval between leaf expansion and bract opening was not predictable, neither based on the half-month period of leaf expansion nor within or among accessions. The data was collected from December 2018 to February 2020 in Araponga, MG, Brazil.
(EPS)
