# Supplementary figures and images for: Synchronized by photoperiod: Shedding light on flowering seasonality in Acrocomia species (Arecaceae)
Source: PLoS One. 2026 Jul 16;21(7):e0352981. doi: 10.1371/journal.pone.0352981 (PMC13374979; doi:10.1371/journal.pone.0352981)

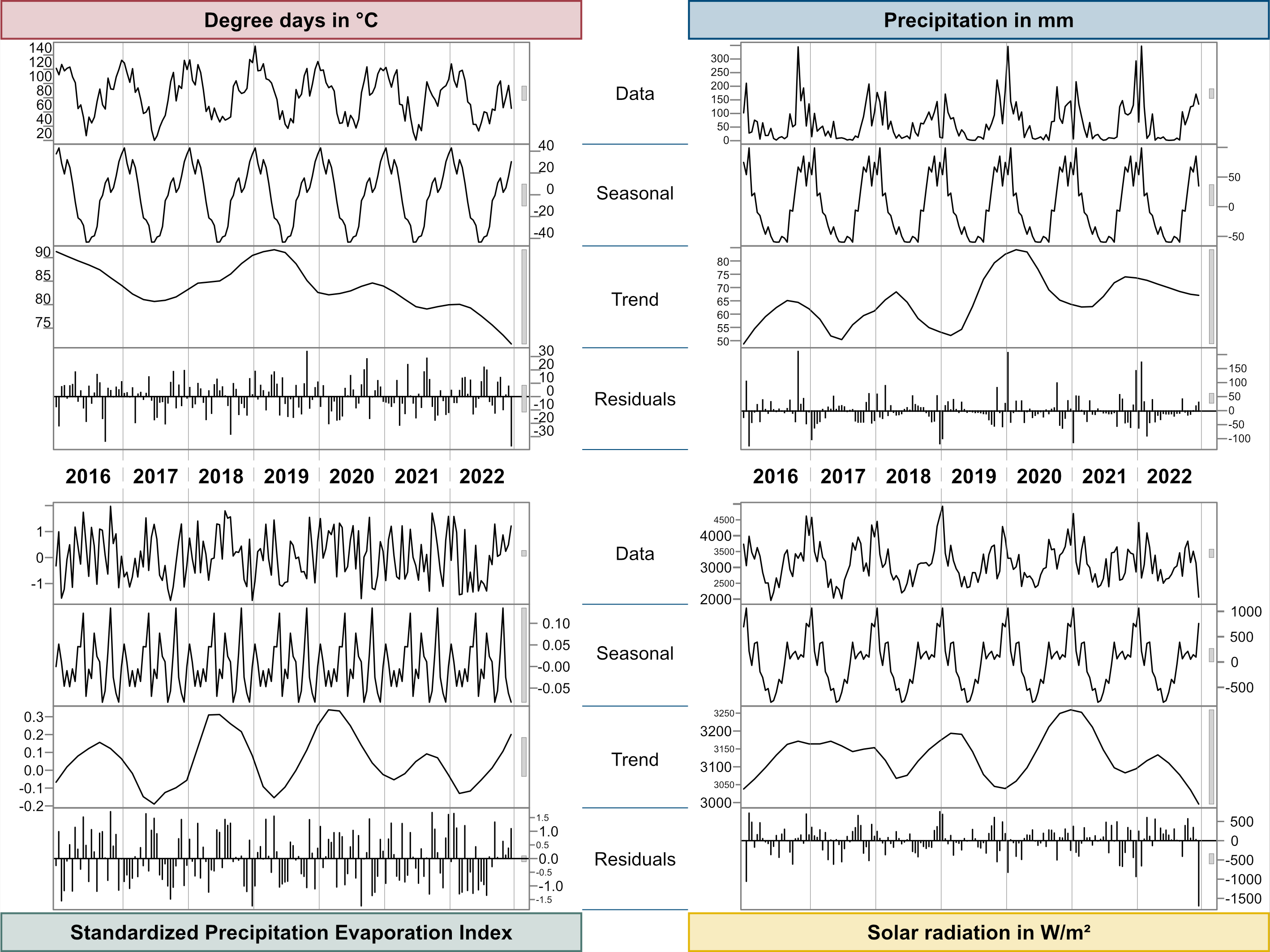

Supplement: S1 Fig — (TIF) [file pone.0352981.s001.tif]

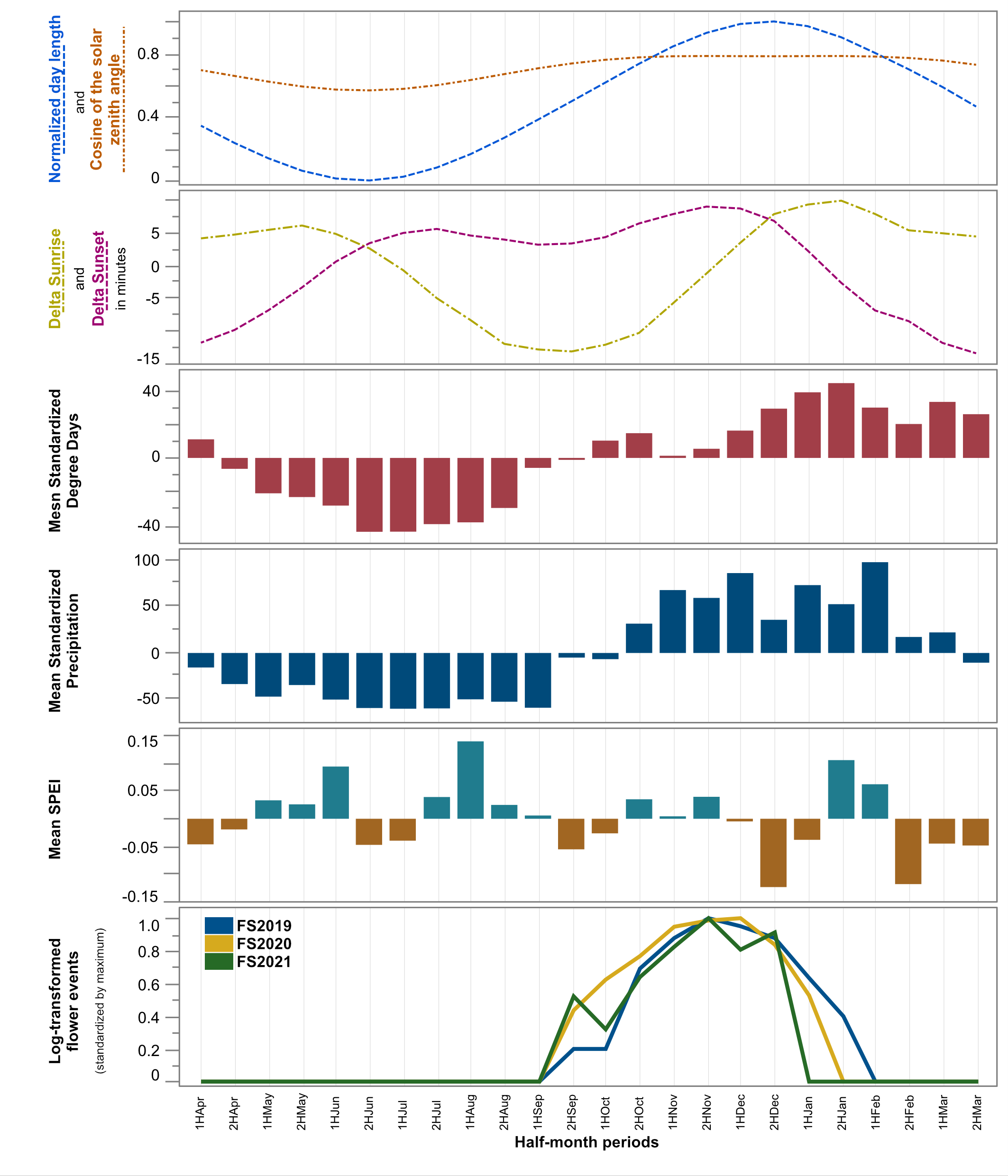

Supplement: S2 Fig — (TIF) [file pone.0352981.s002.tif]

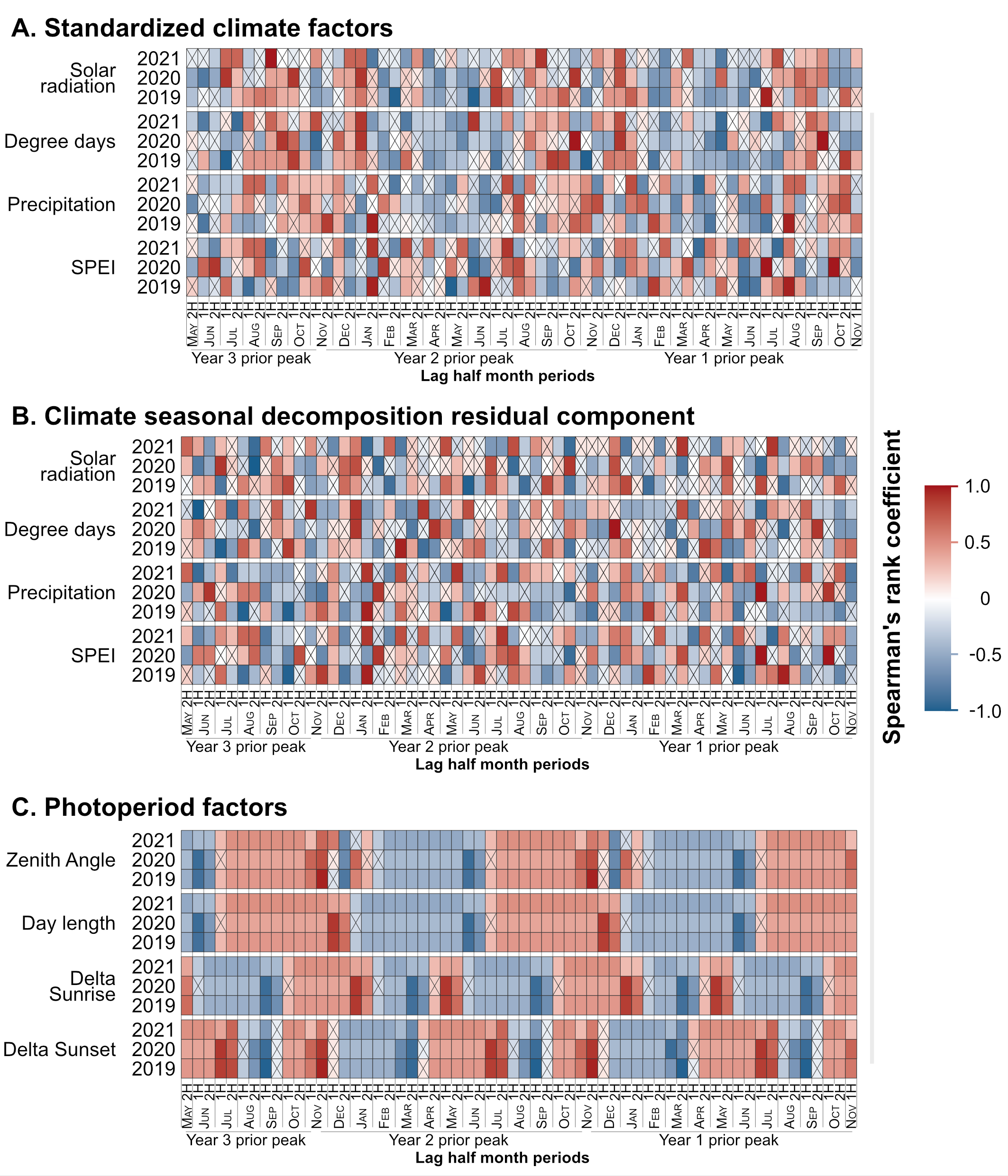

Supplement: S3 Fig — The x-axis represents half-month periods within a single year, beginning after the fall equinox in 2HMar. The panels show photoperiod-related variables, and mean values of standardized degree days, standardized precipitation, and SPEI for Araponga, MG, Brazil, averaged per half-month across 2017–2022. For each year, climate data were previously aggregated following the methodology described in the Material and Methods section. Log-transformed counts of flowering events are plotted on the same time scale to illustrate seasonal dynamics in relation to climatic and photoperiodic variation. (TIF) [file pone.0352981.s003.tif]

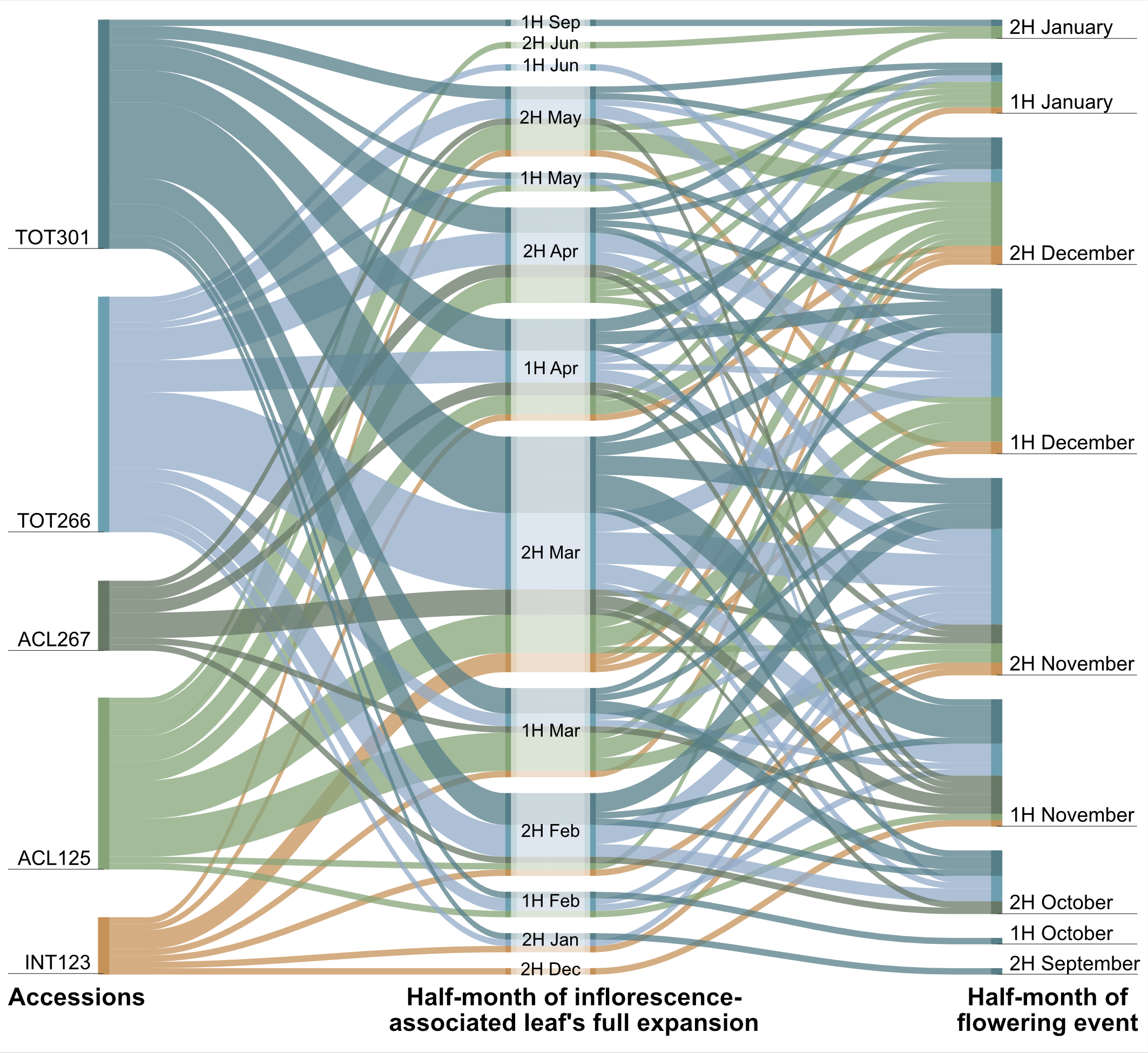

Supplement: S4 Fig — The indicated half-month corresponds to the middle of the moving correlation window. Periods showing non-significant correlations, based on the confidence intervals are crossed out. (TIF) [file pone.0352981.s004.tif]

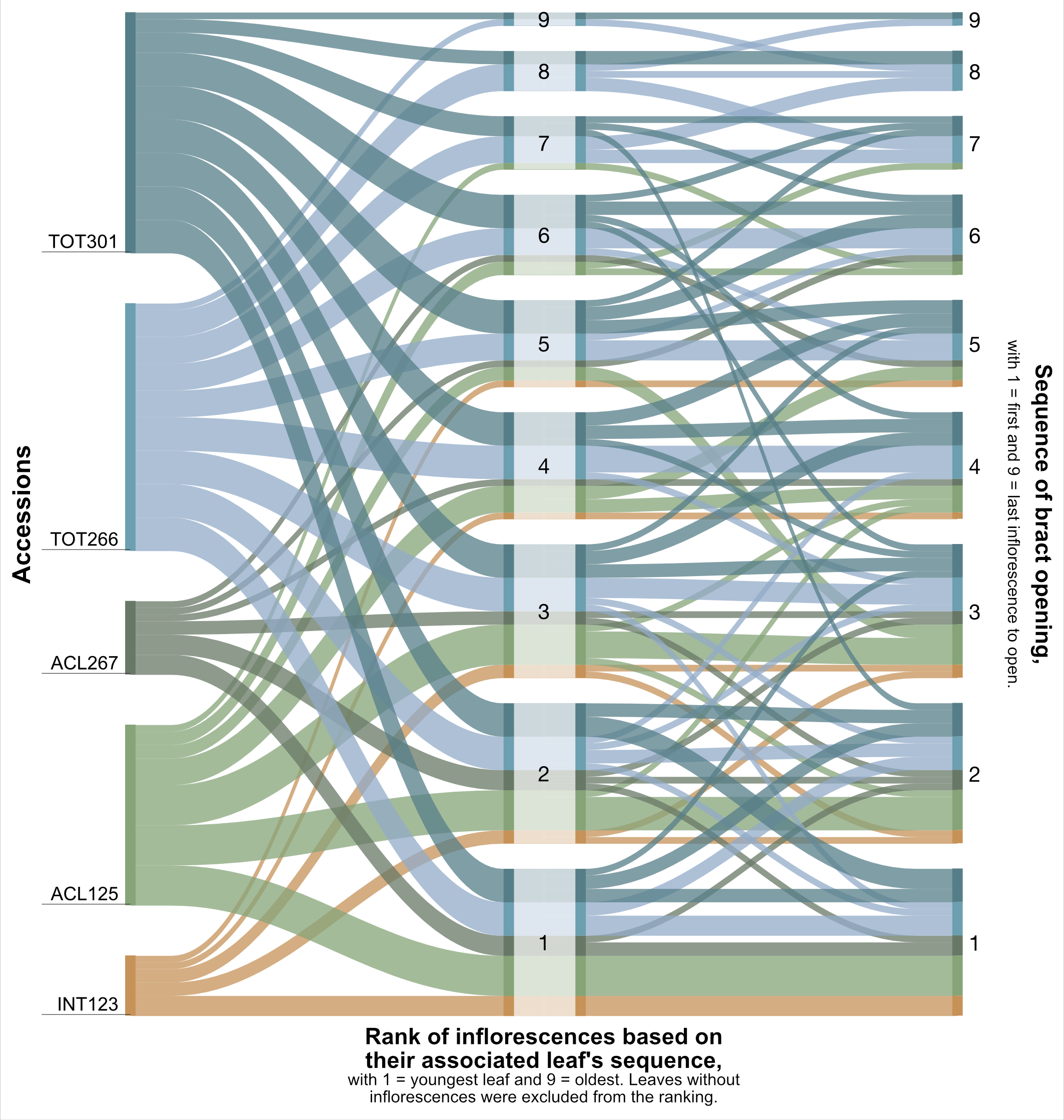

Supplement: S5 Fig — Inflorescences do not necessarily open in relation to the age of their associated leaf. Data were collected during the FS2019 in Araponga, MG, Brazil. (TIF) [file pone.0352981.s005.tif]
