# Supplementary material for: Synchronized by photoperiod: Shedding light on flowering seasonality in Acrocomia species (Arecaceae)
Source: PLoS One. 2026 Jul 16;21(7):e0352981. doi: 10.1371/journal.pone.0352981 (PMC13374979; doi:10.1371/journal.pone.0352981)
Supplement: Attachment — Submitted filename: Response to Reviewers.pdf [file pone.0352981.s009.pdf]

UNIVERSITY OF HOHENHEIM
University of Hohenheim (490e) | 70593 Stuttgart

Faculty of Agriculture
Hans-Ruthenberg Institute of Agricultural Sciences in the Tropics and Subtropics
Department Agronomy in the Tropics and Subtropics

Ms. Catherine Meyer
M.Sc. Agricultural Sciences
M.Sc. ETHZ Plant Biology
Doctoral Student

E catherine.meyer@uni-hohenheim.de

Stuttgart, 2. May 2026

Dear Reviewers,

We sincerely thank you for the careful evaluation of our manuscript, and for the constructive and insightful comments. We have revised the manuscript accordingly and believe it has been significantly improved.

The major changes in response to your comments include:

(i) The reorganization oft he Discussion to incorporate the comments and to improve coherence between the original and newly added sections.
(ii) Subheadings were integrated into the Discussion
(iii) The title was revised by removing "not rain" to reduce dichotomous framing. We expanded the discussion on the association between precipitation and SPEI two years prior to flowering with the flowering peak. The signal follows the flowering peak, which warranted additional discussion; accordingly, the contrast "Synchronized by photoperiod, not rain" was no longer considered appropriate.
(iv) Minor changes were made to the Abstract, Results, and Conclusion to ensure consistency with the expanded discussion on water availability two years prior to flowering.

Below, we provide a detailed, point-by-point response to all comments:

UNIVERSITY OF HOHENHEIM
Address, Street, No.
70599 Stuttgart
Germany
www.uni-hohenheim.de/english

BADEN-WÜRTTEMBERGISCHE BANK
IBANDE20 6005 0101 0002 5601 08
BIC-Code SOLADEST600
UST-IDDE 147 794 207

DIRECTIONS
Tram
U3, Plieningen (Universität Hohenheim)
Bus
65, 70, 73, 74, 75, 76, 79

Zertifikat seit 2004 audit familiengerechte hochschule

Reviewer #1

Comment 1

Reviewer comment (shortened): The title presents a strong dichotomy given evidence of some precipitation effects; consider a more balanced framing.

Response

The title was revised to "Synchronized by photoperiod: Shedding light on flowering seasonality in *Acrocomia* species (Arecaceae)" and "not rain" was removed to reduce dichotomous framing. The revised title is reflected in the title section of the manuscript and better aligns with the observed weaker and less consistent precipitation effects while maintaining photoperiod as the main focus.

The Discussion was reorganized to distinguish between long-term (new lines 584-611) and short-term (new lines 553-583) environmental cues. Photoperiod is now emphasized as the primary regulator of flowering seasonality, with climatic variation considered a secondary, short-term modulating factor (new lines 507–512). Statistically significant long-term associations with water availability are retained and discussed as likely reflecting developmental processes rather than direct seasonal control (new lines 584–587, 591–594), supported by comparisons with other palm species (new lines 597–608).

Comment 2

Reviewer comment (shortened): Causal interpretation of lagged rainfall/SPEI effects is not justified; clarify association vs causation and add developmental context.

Response:

All sentences implying causality for the long-term climatic associations (former lines 426–433; new lines 561–568) were revised to describe statistical associations only, and causal language was removed.

To contextualize the observed lag structure, the Discussion was expanded with information on inflorescence initiation and development timelines in palms, including *Elaeis guineensis* and *Cocos nucifera* (new lines 568–581), providing biological context for the observed delayed associations without implying causality.

Comment 3

Reviewer comment (shortened): Clarify genotype effects and consistency between abstract and discussion; discuss biological relevance and G×E implications.

Response:

The Abstract was revised to clarify that variation among accessions was an objective rather than a principal finding.

The Discussion now explicitly states that most differences among accessions were not statistically significant and are unlikely to be biologically meaningful (new lines 636-663). The only observed variation concerns angular dispersion, which reflects differences in flowering frequency and timing of events. Short scetions were added to discuss implications for genotype × environment interactions and breeding (new lines 653-663; 675-680;715-719).

Comment 4

Reviewer comment (shortened): "Climate remainder" is not sufficiently defined or interpreted.

Response:

A Methods paragraph was added describing the calculation and ecological meaning of the residual component (lines 331–341). The term "remainder" was replaced with "residual component of the seasonal decomposition" or "residual components" throughout the manuscript (new lines 352, 364, 486, 492, 501; within Fig 8, S1Fig, S4Fig (including captions of all three figures)).

Comment 5

Reviewer comment (shortened): Statements about photoperiod as dominant driver should be geographically and temporally qualified.

Response:

The statement was revised to explicitly restrict inference to the environmental conditions in Araponga during the study period. The revised sentence now reads: "…photoperiod acts as the dominant anticipatory signal regulating seasonality under the conditions in Araponga during the study period…" (new lines 508–512).

Comment 6

Reviewer comment (shortened): Temporal codes are difficult to read; add calendar equivalents.

Response:

Calendar equivalents (e.g. "late January 2017") were added at first mention, with original codes retained in brackets for consistency with figures and tables (new lines 493-494).

Comment 7

Reviewer comment (shortened): Add limitations paragraph.

Response:

A limitations paragraph was added at the end of the first part of the Discussion (new lines 663-679), addressing the single study site, limited number of flowering seasons, and correlational analytical framework, among others.

Comment 8

Reviewer comment (shortened): Expand integration of palm developmental biology.

Response:

The Discussion was expanded to include additional developmental context. This includes interspecific comparisons (lines 566–573), developmental timelines in *Elaeis guineensis* and *Cocos nucifera* (lines 597–607), and inflorescence abortion dynamics in *E. guineensis* (lines 624–627), to better situate the observed lagged climatic associations.

Reviewer #2

Comment 1

Reviewer comment (shortened): Geographic scope may be overstated; references to Cuba are incorrect.

Response:

The reference to Cuba was removed, as the cited study concerns fruiting phenology. The revised text restricts comparisons to regions with available flowering data (Brazil, Colombia, Mexico) and avoids overextension of geographic inference (lines 517-527).

Comment 2

Reviewer comment (shortened): Add latitude references to Figure 2.

Response:

The Equator and Tropic of Capricorn were added to Figure 2. The Tropic of Cancer lies outside the map extent.

Comment 3

Reviewer comment (shortened): few minor changes are noted in the table below.

Response:

We thank the reviewer. All suggested minor edits were implemented as indicated in the response table.

Place/Line	Text	Comment	Response
59	Mauritia armata	Change to Mauritiella armata	Was changed
85	Carreño-Barrera (2021)	Carreño-Barrera et al. (2021)	Was changed
105	Drives	Change to drive	Was changed
113-117		Eliminate this paragraph. No need to resume results in the introduction.	Was accepted and deleted

145	in the case of *Acrocomia*	Delete or change as needed	It was deleted
147	whereas anthesis starts around 24 hours after bract opening	whereas staminate anthesis …	Was changed
487-488	as reported across the species' range from southwest and central Brazil, Mexico, to Cuba [33,40,74,75]	None of the cited references covers México, Cuba or anywhere in the northern hemisphere	This was changed. Details on the changes are found in our response to the Comment 1 of Reviewer #2
501-502	The response of palms to seasonal change is in daylight hours	The response of palms to seasonal change in daylight hours	Was changed
748		Revise authors' names citations	Author's names of all references were rechecked and corrected if necessary.
Figure 2		As photoperiods are related to latitude, I suggest adding parallels to the map, at least the equator and the tropics of Cancer and Capricorn. This will make evident that most studied accessions come from areas far from the equator	See response to Comment 2 (Reviewer #2)

We hope that the revised manuscript meets the requirements for publication and that our responses adequately address all comments. We remain at the Editor's disposal for any further clarifications.

Sincerely on the behalf of all authors,
Catherine Meyer
