# Supplementary material for: Synchronized by photoperiod: Shedding light on flowering seasonality in Acrocomia species (Arecaceae)
Source: PLoS One. 2026 Jul 16;21(7):e0352981. doi: 10.1371/journal.pone.0352981 (PMC13374979; doi:10.1371/journal.pone.0352981)
Supplement: Attachment — Submitted filename: Acrocomia aculeata phtoperiod.docx [file pone.0352981.s007.docx]

Synchronized by photoperiod, not rain: Shedding light on flowering seasonality in *Acrocomia* species (Arecaceae)

This is a nice contribution, based on an unusually long study period and well-supported statistically.

I just note that, except for an accession of *Acrocomia intumescens* from northeastern Brazil, all studied accessions originate from around 14° south latitude or farther south. Thus, at least for the widely distributed *Acrocomia aculeata*, the reach of the results should be restricted to palms from those latitudes. How would an accession from Mexico, Cuba or equatorial Colombia “behave” at Araponga? We do not know. The statement (lines 486-488) that “*Acrocomia* exhibits high regional synchrony of flowering during periods of increasing daylength, with a peak around the summer solstice, as reported across the species’ range from southwest and central Brazil, Mexico, to Cuba [33,40,74,75]” is misleading, as none of the cited references includes studies in Mexico or Cuba nor in the norhern limit of the species’ range.

In line with this comment, I suggest adding parallels to the map of Fig. 2, at least the equator and the tropics of Cancer and Capricorn. This will make evident that most studied accessions come from areas far from the equator, thus helping to frame the reach of the results.

A few minor changes are noted in the table below.

| **Place/Line** | **Text** | **Comment** |  |
| --- | --- | --- | --- |
| 59 | Mauritia armata | Change to Mauritiella armata |  |
| 85 | Carreño-Barrera (2021) | Carreño-Barrera et al. (2021) |  |
| 105 | Drives | Change to drive |  |
| 113-117 |  | Eliminate this paragraph. No need to resume results in the introduction. |  |
| 145 | in the case of *Acrocomia* | Delete or change as needed |  |
| 147 | whereas anthesis starts around 24 hours after bract opening | whereas staminate anthesis … |  |
| 487-488 | as reported across the species’ range  from southwest and central Brazil, Mexico, to Cuba [33,40,74,75] | None of the cited references covers México, Cuba or anywhere in the northern hemisphere |  |
| 501-502 | The response of palms to seasonal change is in daylight hours | The response of palms to seasonal change in daylight hours |  |
| 748 |  | Revise authors’ names citations |  |
| Figure 2 |  | As photoperiods are related to latitude, I suggest adding parallels to the map, at least the equator and the tropics of Cancer and Capricorn. This will make evident that most studied accessions come from areas far from the equator |  |
