# Supplementary material for: Synchronized by photoperiod: Shedding light on flowering seasonality in Acrocomia species (Arecaceae)
Source: PLoS One. 2026 Jul 16;21(7):e0352981. doi: 10.1371/journal.pone.0352981 (PMC13374979; doi:10.1371/journal.pone.0352981)
Supplement: Peer review history

# Peer review: Decision Letter 0

**DOI:** 10.1371/journal.pone.0352981.r001

**Authors:** Branislav Šiler

2 Mar 2026

PONE-D-25-68233

Synchronized by photoperiod, not rain: Shedding light on flowering seasonality in *Acrocomia*  species (Arecaceae)

PLOS One

Dear Dr. Meyer,

Thank you for submitting your manuscript to PLOS ONE. After careful consideration, we feel that it has merit but does not fully meet PLOS ONE’s publication criteria as it currently stands. Therefore, we invite you to submit a revised version of the manuscript that addresses the points raised during the review process.

==============================

**Both reviewers judged positively on the manuscript content. However, in their reports, they provided constructive advises to further improve its overall quality. Please mind the attached Reviewer #2's table containing additional comments.**

==============================

Please submit your revised manuscript by Apr 16 2026 11:59PM. If you will need more time than this to complete your revisions, please reply to this message or contact the journal office at plosone@plos.org. When you're ready to submit your revision, log on to https://www.editorialmanager.com/pone/ and select the 'Submissions Needing Revision' folder to locate your manuscript file.

Please include the following items when submitting your revised manuscript:

- A letter that responds to each point raised by the academic editor and reviewer(s). You should upload this letter as a separate file labeled 'Response to Reviewers'.
- A marked-up copy of your manuscript that highlights changes made to the original version. You should upload this as a separate file labeled 'Revised Manuscript with Track Changes'.
- An unmarked version of your revised paper without tracked changes. You should upload this as a separate file labeled 'Manuscript'.

-->If you would like to make changes to your financial disclosure, please include your updated statement in your cover letter. Guidelines for resubmitting your figure files are available below the reviewer comments at the end of this letter.

If applicable, we recommend that you deposit your laboratory protocols in protocols.io to enhance the reproducibility of your results. Protocols.io assigns your protocol its own identifier (DOI) so that it can be cited independently in the future. For instructions see: https://journals.plos.org/plosone/s/submission-guidelines#loc-laboratory-protocols. Additionally, PLOS ONE offers an option for publishing peer-reviewed Lab Protocol articles, which describe protocols hosted on protocols.io. Read more information on sharing protocols at https://plos.org/protocols?utm_medium=editorial-email&utm_source=authorletters&utm_campaign=protocols.

We look forward to receiving your revised manuscript.

Kind regards,

Branislav T. Šiler, Ph.D.

Academic Editor

PLOS One

Journal Requirements:

When submitting your revision, we need you to address these additional requirements.

1. Please ensure that your manuscript meets PLOS ONE's style requirements, including those for file naming. The PLOS ONE style templates can be found at

https://journals.plos.org/plosone/s/file?id=wjVg/PLOSOne_formatting_sample_main_body.pdf and

https://journals.plos.org/plosone/s/file?id=ba62/PLOSOne_formatting_sample_title_authors_affiliations.pdf.

2. Please include a complete copy of PLOS’ questionnaire on inclusivity in global research in your revised manuscript. Our policy for research in this area aims to improve transparency in the reporting of research performed outside of researchers’ own country or community. The policy applies to researchers who have travelled to a different country to conduct research, research with Indigenous populations or their lands, and research on cultural artefacts. The questionnaire can also be requested at the journal’s discretion for any other submissions, even if these conditions are not met.  Please find more information on the policy and a link to download a blank copy of the questionnaire here: https://journals.plos.org/plosone/s/best-practices-in-research-reporting. Please upload a completed version of your questionnaire as Supporting Information when you resubmit your manuscript.

3. Thank you for stating in your Funding Statement:

“CM was funded and supported by the Luxembourg National Research Fund (FNR, fnr.lu) through an AFR-outgoing grant (Grant Code 12532302).”

Please provide an amended statement that declares *all* the funding or sources of support (whether external or internal to your organization) received during this study, as detailed online in our guide for authors at http://journals.plos.org/plosone/s/submit-now.  Please also include the statement “There was no additional external funding received for this study.” in your updated Funding Statement.

Please include your amended Funding Statement within your cover letter. We will change the online submission form on your behalf.

4. Thank you for stating the following financial disclosure:

“CM was funded and supported by the Luxembourg National Research Fund (FNR, fnr.lu) through an AFR-outgoing grant (Grant Code 12532302).”

Please state what role the funders took in the study.  If the funders had no role, please state: "The funders had no role in study design, data collection and analysis, decision to publish, or preparation of the manuscript."

If this statement is not correct you must amend it as needed.

Please include this amended Role of Funder statement in your cover letter; we will change the online submission form on your behalf.

5. We note that you have included the phrase “data not shown” in your manuscript. Unfortunately, this does not meet our data sharing requirements. PLOS does not permit references to inaccessible data. We require that authors provide all relevant data within the paper, Supporting Information files, or in an acceptable, public repository. Please add a citation to support this phrase or upload the data that corresponds with these findings to a stable repository (such as Figshare or Dryad) and provide and URLs, DOIs, or accession numbers that may be used to access these data. Or, if the data are not a core part of the research being presented in your study, we ask that you remove the phrase that refers to these data.

6. We note that Figure 2 in your submission contain [map/satellite] images which may be copyrighted. All PLOS content is published under the Creative Commons Attribution License (CC BY 4.0), which means that the manuscript, images, and Supporting Information files will be freely available online, and any third party is permitted to access, download, copy, distribute, and use these materials in any way, even commercially, with proper attribution. For these reasons, we cannot publish previously copyrighted maps or satellite images created using proprietary data, such as Google software (Google Maps, Street View, and Earth). For more information, see our copyright guidelines: http://journals.plos.org/plosone/s/licenses-and-copyright.

We require you to either (1) present written permission from the copyright holder to publish these figures specifically under the CC BY 4.0 license, or (2) remove the figures from your submission:

a. You may seek permission from the original copyright holder of Figure 2 to publish the content specifically under the CC BY 4.0 license.

We recommend that you contact the original copyright holder with the Content Permission Form (http://journals.plos.org/plosone/s/file?id=7c09/content-permission-form.pdf) and the following text:

“I request permission for the open-access journal PLOS ONE to publish XXX under the Creative Commons Attribution License (CCAL) CC BY 4.0 (http://creativecommons.org/licenses/by/4.0/). Please be aware that this license allows unrestricted use and distribution, even commercially, by third parties. Please reply and provide explicit written permission to publish XXX under a CC BY license and complete the attached form.”

Please upload the completed Content Permission Form or other proof of granted permissions as an "Other" file with your submission.

In the figure caption of the copyrighted figure, please include the following text: “Reprinted from [ref] under a CC BY license, with permission from [name of publisher], original copyright [original copyright year].”

b. If you are unable to obtain permission from the original copyright holder to publish these figures under the CC BY 4.0 license or if the copyright holder’s requirements are incompatible with the CC BY 4.0 license, please either i) remove the figure or ii) supply a replacement figure that complies with the CC BY 4.0 license. Please check copyright information on all replacement figures and update the figure caption with source information. If applicable, please specify in the figure caption text when a figure is similar but not identical to the original image and is therefore for illustrative purposes only.

The following resources for replacing copyrighted map figures may be helpful:

USGS National Map Viewer (public domain): http://viewer.nationalmap.gov/viewer/

The Gateway to Astronaut Photography of Earth (public domain): http://eol.jsc.nasa.gov/sseop/clickmap/

Maps at the CIA (public domain): https://www.cia.gov/library/publications/the-world-factbook/index.html and https://www.cia.gov/library/publications/cia-maps-publications/index.html

NASA Earth Observatory (public domain): http://earthobservatory.nasa.gov/

Landsat: http://landsat.visibleearth.nasa.gov/

USGS EROS (Earth Resources Observatory and Science (EROS) Center) (public domain): http://eros.usgs.gov/#

Natural Earth (public domain): http://www.naturalearthdata.com/

7. If the reviewer comments include a recommendation to cite specific previously published works, please review and evaluate these publications to determine whether they are relevant and should be cited. There is no requirement to cite these works unless the editor has indicated otherwise.

8. Please review your reference list to ensure that it is complete and correct. If you have cited papers that have been retracted, please include the rationale for doing so in the manuscript text, or remove these references and replace them with relevant current references. Any changes to the reference list should be mentioned in the rebuttal letter that accompanies your revised manuscript. If you need to cite a retracted article, indicate the article’s retracted status in the References list and also include a citation and full reference for the retraction notice.

[Note: HTML markup is below. Please do not edit.]

Reviewers' comments:

Reviewer's Responses to Questions

**Comments to the Author**

1. Is the manuscript technically sound, and do the data support the conclusions?

The manuscript must describe a technically sound piece of scientific research with data that supports the conclusions. Experiments must have been conducted rigorously, with appropriate controls, replication, and sample sizes. The conclusions must be drawn appropriately based on the data presented.

Reviewer #1: Yes

Reviewer #2: Yes

**********

2. Has the statistical analysis been performed appropriately and rigorously?

Reviewer #1: Yes

Reviewer #2: Yes

**********

3. Have the authors made all data underlying the findings in their manuscript fully available?

The PLOS Data policy requires authors to make all data underlying the findings described in their manuscript fully available without restriction, with rare exception (please refer to the Data Availability Statement in the manuscript PDF file). The data should be provided as part of the manuscript or its supporting information, or deposited to a public repository. For example, in addition to summary statistics, the data points behind means, medians and variance measures should be available. If there are restrictions on publicly sharing data—e.g. participant privacy or use of data from a third party—those must be specified.

Reviewer #1: Yes

Reviewer #2: Yes

**********

4. Is the manuscript presented in an intelligible fashion and written in standard English?

PLOS ONE does not copyedit accepted manuscripts, so the language in submitted articles must be clear, correct, and unambiguous. Any typographical or grammatical errors should be corrected at revision, so please note any specific errors here.

Reviewer #1: Yes

Reviewer #2: Yes

**********

5. Review Comments to the Author

Please use the space provided to explain your answers to the questions above. You may also include additional comments for the author, including concerns about dual publication, research ethics, or publication ethics. (Please upload your review as an attachment if it exceeds 20,000 characters)

Reviewer #1: Reviewer Report – PONE-D-25-68233: Synchronized by photoperiod, not rain: Shedding light on flowering seasonality in Acrocomia species (Arecaceae)

This manuscript presents a well-designed and methodologically robust analysis of flowering phenology in Acrocomia accessions cultivated under common garden conditions. The application of circular statistics combined with seasonal decomposition and moving-window correlations is appropriate and innovative for phenological research. The study addresses an important gap regarding environmental drivers of flowering in a genus with increasing relevance for sustainable oilseed production.

The results convincingly show strong seasonality and consistent synchronization across accessions and years, with photoperiod emerging as the predominant environmental correlate. The manuscript is generally well written, logically structured, and supported by adequate statistical analyses.However, several points require clarification and moderate revision before the manuscript is suitable for publication.

The manuscript presents a valuable and methodologically sound contribution to tropical phenology and crop domestication research. The main findings are clear and relevant. I recommend minor to moderate revision to address the interpretative and framing issues. Once revised, this study will provide meaningful insight into environmental regulation of flowering in Acrocomia and its implications for breeding and cultivation strategies.

Major Comments

• The title and short title present a strong dichotomy. However, the Results and Discussion acknowledge statistically significant correlations with precipitation and SPEI at specific lag periods (lines 426–433; 456–460). While photoperiod clearly appears dominant, precipitation effects, although weaker and less consistent, are not entirely absent. Consider moderating the framing to reflect that flowering is primarily regulated by photoperiod but potentially fine-tuned by episodic climatic variation. This would better align the title with the presented evidence.

• The manuscript suggests that rainfall or drought anomalies two years prior “influence” reproductive development (lines 426–433). However, moving-window correlations cannot establish causality. Please clarify that these findings represent statistical associations rather than demonstrated causal mechanisms. The Discussion would benefit from a short paragraph outlining known timelines of inflorescence initiation and development in palms to support (or cautiously contextualize) the proposed biological interpretation.

• The abstract highlights variation among accessions, yet the Discussion emphasizes low inter-annual variation and strong synchrony (lines 441–443). The manuscript would benefit from clearer interpretation of genotype effects. Clarify whether statistical differences among accessions were significant and biologically meaningful. Briefly discuss implications for genotype x environment interactions and breeding strategies, Especially regarding transferability across latitudes.

• The term “climate remainder” is used in the Results and Discussion (lines 423–424; 435–437), but its biological interpretation is not sufficiently explained. Expand the Methods section to clearly define how the remainder was calculated (ex. residuals after seasonal decomposition) and explain its ecological relevance in the context of phenological triggers.

• Statements such as “photoperiod is the dominant driver” (lines 440–443) should be qualified to indicate that conclusions are based on conditions in Araponga and the studied seasons.

• Although defined in the Methods, expressions such as “2HJan2017” may be difficult for readers. Consider adding calendar equivalents (ex., “late January 2017”) at first mention in the Results.

• A brief paragraph acknowledging limitations would strengthen the manuscript. For example: (1) Single experimental site, (2) just three flowering seasons and, (3) correlational analytical framework

• The Discussion would benefit from deeper integration of palm developmental biology (ex. timing of inflorescence differentiation relative to environmental cues).

Reviewer #2: This is a nice contribution, based on an unusually long study period and well-supported statistically.

I just note that, except for an accession of Acrocomia intumescens from northeastern Brazil, all studied accessions originate from around 14° south latitude or farther south. Thus, at least for the widely distributed Acrocomia aculeata, the reach of the results should be restricted to palms from those latitudes. How would an accession from Mexico, Cuba or equatorial Colombia “behave” at Araponga? We do not know. The statement (lines 486-488) that “Acrocomia exhibits high regional synchrony of flowering during periods of increasing daylength, with a peak around the summer solstice, as reported across the species’ range from southwest and central Brazil, Mexico, to Cuba [33,40,74,75]” is misleading, as none of the cited references includes studies in Mexico or Cuba nor in the norhern limit of the species’ range.

In line with this comment, I suggest adding parallels to the map of Fig. 2, at least the equator and the tropics of Cancer and Capricorn. This will make evident that most studied accessions come from areas far from the equator, thus helping to frame the reach of the results.

A few minor changes are noted in the table below.

**********

6. PLOS authors have the option to publish the peer review history of their article (what does this mean?). If published, this will include your full peer review and any attached files.

If you choose “no”, your identity will remain anonymous but your review may still be made public.

**Do you want your identity to be public for this peer review?**  For information about this choice, including consent withdrawal, please see our Privacy Policy.

Reviewer #1: **Yes:**  JONATHAN A. MORALES-MARROQUIN

Reviewer #2: No

**********

[NOTE: If reviewer comments were submitted as an attachment file, they will be attached to this email and accessible via the submission site. Please log into your account, locate the manuscript record, and check for the action link "View Attachments". If this link does not appear, there are no attachment files.]

To ensure your figures meet our technical requirements, please review our figure guidelines: https://journals.plos.org/plosone/s/figures

You may also use PLOS’s free figure tool, NAAS, to help you prepare publication quality figures: https://journals.plos.org/plosone/s/figures#loc-tools-for-figure-preparation.

NAAS will assess whether your figures meet our technical requirements by comparing each figure against our figure specifications.

Attachment Submitted filename: Acrocomia aculeata phtoperiod.docx

# Author comment: Author response to Decision Letter 1

**DOI:** 10.1371/journal.pone.0352981.r002

16 May 2026

Reviewer #1

Comment 1

Reviewer comment (shortened): The title presents a strong dichotomy given evidence of some precipitation effects; consider a more balanced framing.

Response

The title was revised to “Synchronized by photoperiod: Shedding light on flowering seasonality in Acrocomia species (Arecaceae)” and “not rain” was removed to reduce dichotomous framing. The revised title is reflected in the title section of the manuscript and better aligns with the observed weaker and less consistent precipitation effects while maintaining photoperiod as the main focus.

The Discussion was reorganized to distinguish between long-term (new lines 584-611) and short-term (new lines 553-583) environmental cues. Photoperiod is now emphasized as the primary regulator of flowering seasonality, with climatic variation considered a secondary, short-term modulating factor (new lines 507–512). Statistically significant long-term associations with water availability are retained and discussed as likely reflecting developmental processes rather than direct seasonal control (new lines 584–587, 591–594), supported by comparisons with other palm species (new lines 597–608).

Comment 2

Reviewer comment (shortened): Causal interpretation of lagged rainfall/SPEI effects is not justified; clarify association vs causation and add developmental context.

Response:

All sentences implying causality for the long-term climatic associations (former lines 426–433; new lines 561–568) were revised to describe statistical associations only, and causal language was removed.

To contextualize the observed lag structure, the Discussion was expanded with information on inflorescence initiation and development timelines in palms, including Elaeis guineensis and Cocos nucifera (new lines 568–581), providing biological context for the observed delayed associations without implying causality.

Comment 3

Reviewer comment (shortened): Clarify genotype effects and consistency between abstract and discussion; discuss biological relevance and G×E implications.

Response:

The Abstract was revised to clarify that variation among accessions was an objective rather than a principal finding.

The Discussion now explicitly states that most differences among accessions were not statistically significant and are unlikely to be biologically meaningful (new lines 636-663). The only observed variation concerns angular dispersion, which reflects differences in flowering frequency and timing of events. Short scetions were added to discuss implications for genotype × environment interactions and breeding (new lines 653-663; 675-680;715-719).

Comment 4

Reviewer comment (shortened): “Climate remainder” is not sufficiently defined or interpreted.

Response:

A Methods paragraph was added describing the calculation and ecological meaning of the residual component (lines 331–341). The term “remainder” was replaced with “residual component of the seasonal decomposition” or “residual components” throughout the manuscript (new lines 352, 364, 486, 492, 501; within Fig 8, S1Fig, S4Fig (including captions of all three figures)).

Comment 5

Reviewer comment (shortened): Statements about photoperiod as dominant driver should be geographically and temporally qualified.

Response:

The statement was revised to explicitly restrict inference to the environmental conditions in Araponga during the study period. The revised sentence now reads: “…photoperiod acts as the dominant anticipatory signal regulating seasonality under the conditions in Araponga during the study period…” (new lines 508–512).

Comment 6

Reviewer comment (shortened): Temporal codes are difficult to read; add calendar equivalents.

Response:

Calendar equivalents (e.g. “late January 2017”) were added at first mention, with original codes retained in brackets for consistency with figures and tables (new lines 493-494).

Comment 7

Reviewer comment (shortened): Add limitations paragraph.

Response:

A limitations paragraph was added at the end of the first part of the Discussion (new lines 663-679), addressing the single study site, limited number of flowering seasons, and correlational analytical framework, among others.

Comment 8

Reviewer comment (shortened): Expand integration of palm developmental biology.

Response:

The Discussion was expanded to include additional developmental context. This includes interspecific comparisons (lines 566–573), developmental timelines in Elaeis guineensis and Cocos nucifera (lines 597–607), and inflorescence abortion dynamics in E. guineensis (lines 624–627), to better situate the observed lagged climatic associations.

Reviewer #2

Comment 1

Reviewer comment (shortened): Geographic scope may be overstated; references to Cuba are incorrect.

Response:

The reference to Cuba was removed, as the cited study concerns fruiting phenology. The revised text restricts comparisons to regions with available flowering data (Brazil, Colombia, Mexico) and avoids overextension of geographic inference (lines 517-527).

Comment 2

Reviewer comment (shortened): Add latitude references to Figure 2.

Response:

The Equator and Tropic of Capricorn were added to Figure 2. The Tropic of Cancer lies outside the map extent.

Comment 3

Reviewer comment (shortened): few minor changes are noted in the table below.

Response:

We thank the reviewer. All suggested minor edits were implemented as indicated in the response table.

Place/Line Text Comment Response

59 Mauritia armata Change to Mauritiella armata Was changed

85 Carreño-Barrera (2021) Carreño-Barrera et al. (2021) Was changed

105 Drives Change to drive Was changed

113-117 Eliminate this paragraph. No need to resume results in the introduction. Was accepted and deleted

145 in the case of Acrocomia Delete or change as needed It was deleted

147 whereas anthesis starts around 24 hours after bract opening whereas staminate anthesis … Was changed

487-488 as reported across the species’ range

from southwest and central Brazil, Mexico, to Cuba [33,40,74,75] None of the cited references covers México, Cuba or anywhere in the northern hemisphere This was changed. Details on the changes are found in our response to the Comment 1 of Reviewer #2

501-502 The response of palms to seasonal change is in daylight hours The response of palms to seasonal change in daylight hours Was changed

748 Revise authors’ names citations Author’s names of all references were rechecked and corrected if necessary.

Figure 2 As photoperiods are related to latitude, I suggest adding parallels to the map, at least the equator and the tropics of Cancer and Capricorn. This will make evident that most studied accessions come from areas far from the equator See response to Comment 2 (Reviewer #2)

Attachment Submitted filename: Response to Reviewers.pdf

# Editor report: Decision Letter 1

**DOI:** 10.1371/journal.pone.0352981.r003

**Authors:** Branislav Šiler, Branislav Šiler

18 Jun 2026

Synchronized by photoperiod: Shedding light on flowering seasonality in Acrocomia species (Arecaceae)

PONE-D-25-68233R1

Dear Dr. Meyer,

We’re pleased to inform you that your manuscript has been judged scientifically suitable for publication and will be formally accepted for publication once it meets all outstanding technical requirements.

Within one week, you’ll receive an e-mail detailing the required amendments. When these have been addressed, you’ll receive a formal acceptance letter and your manuscript will be scheduled for publication.

An invoice will be generated when your article is formally accepted. Please note, if your institution has a publishing partnership with PLOS and your article meets the relevant criteria, all or part of your publication costs will be covered. Please make sure your user information is up-to-date by logging into Editorial Manager at Editorial Manager® and clicking the ‘Update My Information' link at the top of the page. For questions related to billing, please contact billing support.

If your institution or institutions have a press office, please notify them about your upcoming paper to help maximize its impact. If they’ll be preparing press materials, please inform our press team as soon as possible -- no later than 48 hours after receiving the formal acceptance. Your manuscript will remain under strict press embargo until 2 pm Eastern Time on the date of publication. For more information, please contact onepress@plos.org.

Kind regards,

Branislav T. Šiler, Ph.D.

Academic Editor

PLOS One

Additional Editor Comments (optional):

Reviewers' comments:

# Editor report: Acceptance letter

**DOI:** 10.1371/journal.pone.0352981.r004

**Authors:** Branislav Šiler, Branislav Šiler

PONE-D-25-68233R1

PLOS One

Dear Dr. Meyer,

I'm pleased to inform you that your manuscript has been deemed suitable for publication in PLOS One. Congratulations! Your manuscript is now being handed over to our production team.

At this stage, our production department will prepare your paper for publication. This includes ensuring the following:

* All references, tables, and figures are properly cited

* All relevant supporting information is included in the manuscript submission,

* There are no issues that prevent the paper from being properly typeset

You will receive further instructions from the production team, including instructions on how to review your proof when it is ready. Please keep in mind that we are working through a large volume of accepted articles, so please give us a few days to review your paper and let you know the next and final steps.

Lastly, if your institution or institutions have a press office, please let them know about your upcoming paper now to help maximize its impact. If they'll be preparing press materials, please inform our press team within the next 48 hours. Your manuscript will remain under strict press embargo until 2 pm Eastern Time on the date of publication. For more information, please contact onepress@plos.org.

You will receive an invoice from PLOS for your publication fee after your manuscript has reached the completed accept phase. If you receive an email requesting payment before acceptance or for any other service, this may be a phishing scheme. Learn how to identify phishing emails and protect your accounts at https://explore.plos.org/phishing.

If we can help with anything else, please email us at customercare@plos.org.

Thank you for submitting your work to PLOS ONE and supporting open access.

Kind regards,

PLOS ONE Editorial Office Staff

on behalf of

Dr. Branislav T. Šiler

Academic Editor

PLOS One
